# Quaking promotes monocyte differentiation into pro-atherogenic macrophages by controlling pre-mRNA splicing and gene expression

**DOI:** 10.1038/ncomms10846

**Published:** 2016-03-31

**Authors:** Ruben G. de Bruin, Lily Shiue, Jurriën Prins, Hetty C. de Boer, Anjana Singh, W. Samuel Fagg, Janine M. van Gils, Jacques M. G. J. Duijs, Sol Katzman, Adriaan O. Kraaijeveld, Stefan Böhringer, Wai Y. Leung, Szymon M. Kielbasa, John P. Donahue, Patrick H.J. van der Zande, Rick Sijbom, Carla M. A. van Alem, Ilze Bot, Cees van Kooten, J. Wouter Jukema, Hilde Van Esch, Ton J. Rabelink, Hilal Kazan, Erik A. L. Biessen, Manuel Ares Jr., Anton Jan van Zonneveld, Eric P. van der Veer

**Affiliations:** 1 Einthoven Laboratory of Experimental Vascular Medicine, Leiden University Medical Center, Albinusdreef 2, 2300RC Leiden, The Netherlands; 2 Department of Internal Medicine (Nephrology), Leiden University Medical Center, Albinusdreef 2, C7-36, PO Box 9600, 2300RC, Leiden The Netherlands; 3 Center for Molecular Biology of RNA, Department of Molecular, Cell and Developmental Biology, University of California, 1156 High Street, Santa Cruz, California 95064, USA; 4 Department of Pathology, CARIM, Academic University Hospital Maastricht, P. Debyelaan 25, 6229HX Maastricht, The Netherlands; 5 Department of Cardiology, Leiden University Medical Center, Albinusdreef 2, 2300RC Leiden, The Netherlands; 6 Department of Medical Biostatistics, Leiden University Medical Center, Albinusdreef 2, 2300RC Leiden, The Netherlands; 7 Department of Sequencing Analysis Support Core, Leiden University Medical Center, Albinusdreef 2, 2300RC Leiden, The Netherlands; 8 Division of Biopharmaceutics, Leiden/Amsterdam Center for Drug Research, Leiden University, Einsteinweg 55, 2333CC Leiden, The Netherlands; 9 Durrer Center for Cardiogenetic Research, Meiburgdreef 9, 1105AZ Amsterdam, The Netherlands; 10 Department of Human Genetics, University Hospitals Leuven, Herestraat 43, 3000 Leuven, Belgium; 11 Department of Computer Engineering, Antalya International University, Universit Cad. No.2, Antalya 07190, Turkey

## Abstract

A hallmark of inflammatory diseases is the excessive recruitment and influx of monocytes to sites of tissue damage and their ensuing differentiation into macrophages. Numerous stimuli are known to induce transcriptional changes associated with macrophage phenotype, but posttranscriptional control of human macrophage differentiation is less well understood. Here we show that expression levels of the RNA-binding protein Quaking (QKI) are low in monocytes and early human atherosclerotic lesions, but are abundant in macrophages of advanced plaques. Depletion of QKI protein impairs monocyte adhesion, migration, differentiation into macrophages and foam cell formation *in vitro* and *in vivo*. RNA-seq and microarray analysis of human monocyte and macrophage transcriptomes, including those of a unique QKI haploinsufficient patient, reveal striking changes in QKI-dependent messenger RNA levels and splicing of RNA transcripts. The biological importance of these transcripts and requirement for QKI during differentiation illustrates a central role for QKI in posttranscriptionally guiding macrophage identity and function.

## Abstract (web summary)

Post-transcriptional control of RNA is important in health and disease. Here, the authors show that the RNA-binding protein Quaking guides pre-mRNA splicing and transcript abundance during monocyte to macrophage differentiation, and that Quaking depletion impairs pro-atherogenic foam cell formation.

## Graphical abstract

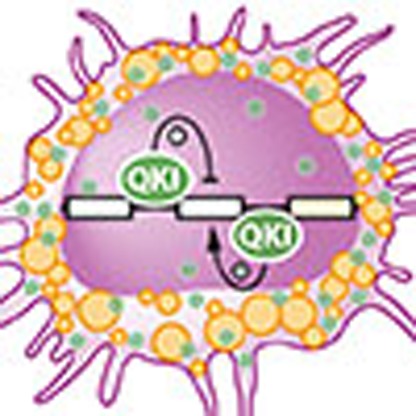

Monocytes serve as danger sensors within the circulation. The activation of blood-borne monocytes by inflammatory stimuli triggers their adhesion and homing to sites of tissue injury, where they differentiate into macrophages and collectively aid in the resolution of damage^1^,^2^. However, the chronic accumulation of macrophages at these sites of injury is a hallmark of inflammatory diseases such as rheumatoid arthritis^3^, Crohn's disease^4^ and atherosclerosis^5^,^6^,^7^.

Dynamic changes in gene expression are associated with monocyte to macrophage differentiation, where PU.1 (ref. 8), Signal Transducer and Activator of Transcription (STATs)^9^ and CCAAT/Enhancer Binding Protein (C/EBP)s^10^ are key transcription factors that drive this alteration in cellular phenotype and function^11^,^12^. Importantly, numerous studies have identified critical roles for both microRNAs (miRNAs) and RNA-binding proteins (RBPs) in posttranscriptionally regulating monocyte^13^ and macrophage^14^ biology. However, the posttranscriptional regulation of monocyte to macrophage differentiation has generally been limited to studies detailing miRNA-based targeting of individual transcription factors or effector molecules that either stimulate or delay this phenotypic conversion^15^,^16^,^17^.

In contrast to miRNAs, RBPs mediate both quantitative and qualitative changes to the transcriptome, interacting with pre-mRNAs to influence (alternative) splicing, transcript stability, editing, subcellular localization and translational activation or repression^18^,^19^,^20^. This broad arsenal of RNA-based control points enables RBPs to modulate the proteome in response to immunogenic stimuli^17^, shifting inflammatory cells from an immature or naive state to a mature or activated state, as has previously been established in lymphoid cells^21^,^22^. In recent times, we discovered that expression of the RBP Quaking (QKI) is induced in human restenotic lesion-resident vascular smooth muscle cells (VSMCs), where it directly mediates a splicing event in the Myocardin pre-mRNA that governs VSMC function^23^. This finding prompted us to investigate whether QKI could similarly serve as an inflammation-sensitive posttranscriptional guide during monocyte to macrophage differentiation. Alternative splicing of the QKI pre-mRNA yields mature transcripts of 5, 6 or 7 kb that encode distinct protein isoforms, namely QKI-5, -6 and -7 (refs 24, 25). QKI-5 possesses a nuclear localization signal in the carboxy-terminal region and is found in the nucleus of cells. In contrast, QKI-6 and QKI-7 are found in the cytoplasm. However, QKI-6 and QKI-7 can also translocate to the nucleus^23^,^26^. The presence of a KH-family homology domain confers QKI with the capacity to bind RNA^27^, albeit dimerization is required^26^,^28^ to bind with high affinity to the QKI response element (QRE) sequence (NACUAAY N1-20 UAAY) on target RNAs^29^,^30^,^31^,^32^,^33^. Importantly, aberrant QKI expression is associated with inflammatory diseases such as schizophrenia^34^,^35^, cancer^36^ and restenosis^23^.

Here we show that the RBP QKI plays a critical role in regulating the conversion of monocytes into macrophages in, for example, atherosclerotic lesions. Our studies pinpoint QKI as a dynamic regulator of pre-mRNA splicing and expression profiles that drive monocyte activation, adhesion and differentiation into macrophages, and facilitates their conversion into foam cells.

## Results

### Human atherosclerotic lesion macrophages express QKI

**Figure 1:**
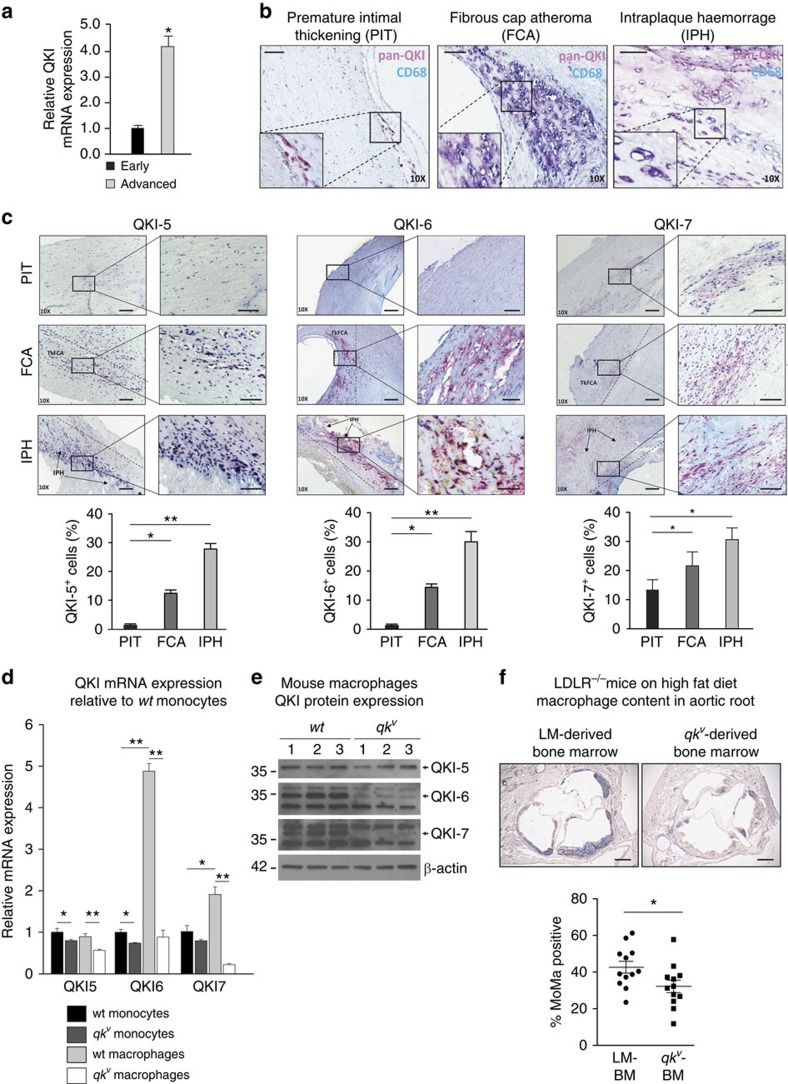
Quaking is expressed in macrophages within atherosclerotic lesions. (**a**) Pan-QKI mRNA expression levels in CD68^+^ macrophages of early and advanced atherosclerotic lesions isolated by laser-capture microdissection (*n*=4). Data expressed as mean±s.e.m.; Student's *t*-test, *P<0.05. Scale bar, 50 μm. (**b**) Immunohistochemical analysis of co-localization of pan-QKI and CD68 expression in preliminary intimal thickening (PIT), FCA and intraplaque haemorrage (PIH). Dashed line denotes intimal/adventitial border. Scale bar, 50 μm. (**c**) Immunohistochemical analysis of QKI-5, -6 and -7 expression in PIT, FCA and IPH (top), and quantification of QKI-positive cells mm^2^ per tissue sample (*n*=5). Data expressed as mean±s.e.m.; one-way analysis of variance (ANOVA), Bonferroni's *post-hoc* test; *P<0.05, **P<0.01. (**d**) Quantitative RT–PCR (qRT–PCR) analysis of QKI mRNA expression in naive BM-derived CD115+ mouse monocytes and 7 days M-CSF stimulated macrophages of either *wt*-littermates (LM) or quaking viable (*qk*^*v*^) mice (*n*=at least 3 mice per condition). Data expressed as mean±s.e.m.; one-way ANOVA, Bonferroni's *post-hoc* test; *P<0.05 and **P<0.01. (**e**) Western blot analysis of QKI-5, -6 and -7 expression levels in 7 days M-CSF stimulated macrophages derived from BM of *wt* and *qk*^*v*^ mice. Each lane represents an individual mouse lysate (biological *n*=3). (**f**) Immunohistochemical analysis for atherosclerotic plaque-resident macrophages (% MoMa-positive area) in aortic root sections of γ-irradiated (8 Gy) *LDLR*^*−*/*−*^ mice that subsequently were transplanted with BM from either *qk*^*v*^ mice (*qk*^*v*^-BM) or littermates (LM)(LM-BM) and fed a high-fat diet for 8 weeks to develop atherosclerotic lesions (*n*=12 per group). Scale bar, 200 μm. Data expressed as mean±s.e.m.; Student's *t*-test, with *P<0.05.

We previously observed QKI expression in VSMCs^23^ and in leukocyte foci within human coronary restenotic lesions. Based on this observation, we used laser-capture microdissection to harvest CD68^+^ macrophages from early and advanced atherosclerotic lesions of human carotid arteries. QKI mRNA was 4.2-fold enriched in macrophages derived from advanced as compared with early atherosclerotic lesions (Fig. 1a). Next, using immunohistochemistry, we assessed QKI protein expression in human tissue sections at various stages of atherosclerotic lesion development, namely early pathological intimal thickening (PIT), fibrous cap atheroma (FCA) and intraplaque haemorrhaging (IPH). Although QKI was detectable in CD68^+^ myeloid cells of PIT, it was abundantly expressed in macrophage-rich FCA and IPH lesions (Fig. 1b). Furthermore, QKI-5, -6 and -7 were detectable in the nuclear, perinuclear and cytoplasmic regions of intimal macrophages in both FCA and IPH, respectively (Fig. 1c). We conclude that the accumulation of macrophages in human atherosclerotic lesions is associated with increased mRNA and protein expression of all three QKI isoforms within the macrophage.

### A reduction in QKI decreases lesional macrophage burden

To investigate whether decreased QKI expression in monocytes and macrophages could influence atherosclerotic lesion formation, we transplanted bone marrow (BM) from QKI viable (*qk*^*v*^) mice^37^, which express reduced levels of QKI protein, or their wild-type (*wt*) littermate controls (LM) into atherogenic *LDLR*^*−/−*^ mice. Although *qk* knockout mice die as embryos, the *qk*^*v*^ mouse harbours a spontaneous ∼1 Mb deletion in the *QK* promoter region that leads to reduced levels of QKI mRNA and protein^37^. Indeed, macrophage colony-stimulating factor (M-CSF)-mediated conversion of LM and *qk*^*v*^ BM-derived monocytes to macrophages showed subtly reduced QKI-5 mRNA and protein levels, and almost a complete ablation of QKI-6 and -7 protein (Fig. 1d,e). Following BM transplantation, the *LDLR*^*−/−*^/*qk*^*v*^ and *LDLR*^*−/−*^/LM mice were fed a high-fat diet for 8 weeks, to induce atherosclerotic lesion formation. Interestingly, the long-term reduction of QKI expression during haematopoietic reconstitution limited neutrophil and monocytic repopulation (Supplementary Data 1). In keeping with this finding, immunohistochemical analysis of the aortic root revealed significantly decreased monocyte/macrophage content within atherosclerotic lesions of *LDLR*^*−/−*^*/qk*^*v*^ mice (Fig. 1f), a finding that immunohistochemical analysis revealed was independent of plaque size or collagen content. These findings suggested that changes in haematopoietic and monocytic QKI expression could influence the macrophage content of atherosclerotic lesions.

### QKI is induced on monocyte to macrophage differentiation

**Figure 2:**
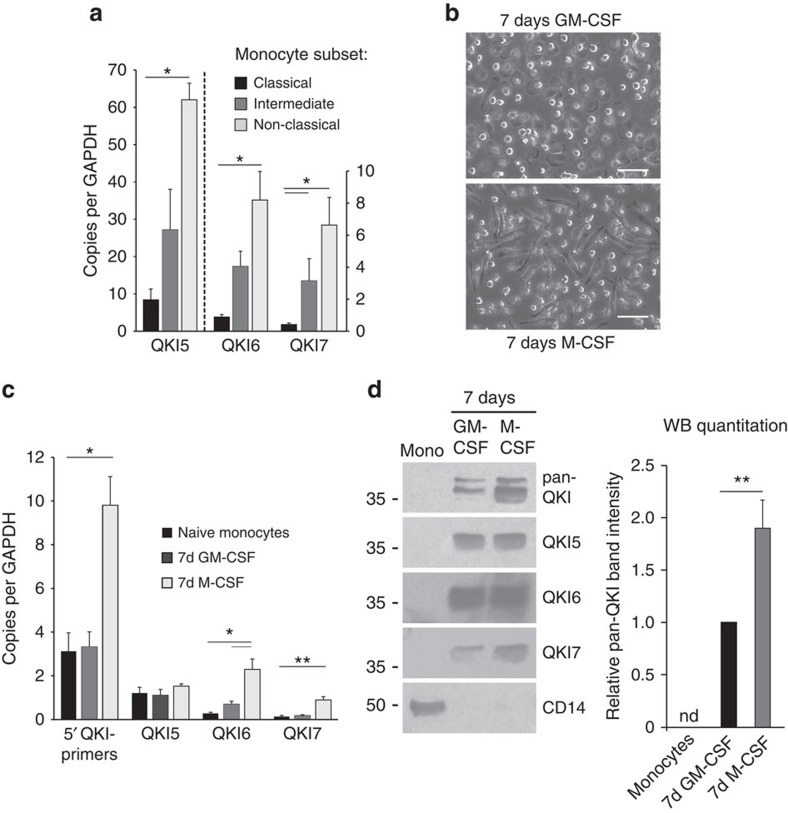
QKI is highly expressed in macrophages derived from PB monocytes. (**a**) mRNA expression levels of distinct QKI isoforms following negative selection and FACS sorting for blood-derived human monocyte subsets, namely classical (CD14^++^, CD16^*−*^), intermediate (CD14^++^,CD16^+^) and non-classical (CD14^+^,CD16^*+*^). Expression is depicted relative to copies per glyceraldehyde 3-phosphate dehydrogenase (GAPDH). Data expressed as mean±s.e.m.; one-way analysis of variance (ANOVA), Bonferroni's *post-hoc* test; *P<0.05 and **P<0.01. (**b**) Phase-contrast photomicrographs of human PB monocytes cultured for 7 days in the presence of either GM-CSF or M-CSF. Scale bar, 50 μm. (**c**) Quantitative RT–PCR (qRT–PCR) analysis for QKI mRNA isoforms in naive PB monocytes isolated using CD14^+^ microbeads, 7 days GM-CSF and 7 days M-CSF differentiated macrophages (*n*=3). Expression is depicted relative to copies per GAPDH. Data expressed as mean±s.e.m.; one-way ANOVA, Bonferroni's *post-hoc* test; *P<0.05 and **P<0.01. (**d**) Western blot analysis of QKI protein isoforms in naive monocytes, 7 days GM-CSF and 7 days M-CSF differentiated macrophages (pan-QKI and CD14: *n*=5, QKI-5, 6 and 7: *n*=1) with quantification of pan-QKI (*n*=5). Data expressed as mean±s.e.m.; Student's *t*-test, with **P<0.01. Equivalent concentrations of whole-cell lysates were loaded per lane as determined using a BCA protein assay.

Having identified high QKI expression in macrophages in atherosclerotic lesions, we first explored whether QKI mRNA expression levels differ in macrophage precursors, namely classical (CD14^++^/CD16^*−*^), intermediate (CD14^++^/CD16^+^) and non-classical (CD14^+^/CD16^+^) monocytes derived from human peripheral blood (PB)^2^. All three monocyte subpopulations abundantly expressed QKI-5, -6 and -7 mRNAs as compared with glyceraldehyde 3-phosphate dehydrogenase (Fig. 2a). Moreover, QKI-5, -6 and -7 mRNA levels increased as classical monocytes progressed towards intermediate or non-classical monocytes. Interestingly, QKI-5 mRNA was the most abundantly expressed transcript in all three subpopulations. (Fig. 2a).

Next, we assessed QKI mRNA and protein levels in human PB monocytes treated with granulocyte–macrophage CSF (GM-CSF) and M-CSF, to stimulate their conversion to pro-inflammatory and anti-inflammatory macrophages, respectively (Fig. 2b). We observed remarkable increases in the expression of all QKI mRNA transcripts in mature macrophages (Fig. 2c). However, despite abundant QKI mRNA, the distinct QKI isoforms were poorly expressed in freshly isolated PB monocytes as compared with mature macrophages (Fig. 2d). The GM-CSF or M-CSF-induced conversion of monocytes into macrophages was associated with striking increases in QKI-5, -6 and -7 protein levels, with a more pronounced increase in all three isoforms observed with M-CSF treatment (Fig. 2d).

### QKI haploinsufficiency perturbs macrophage differentiation

**Figure 3:**
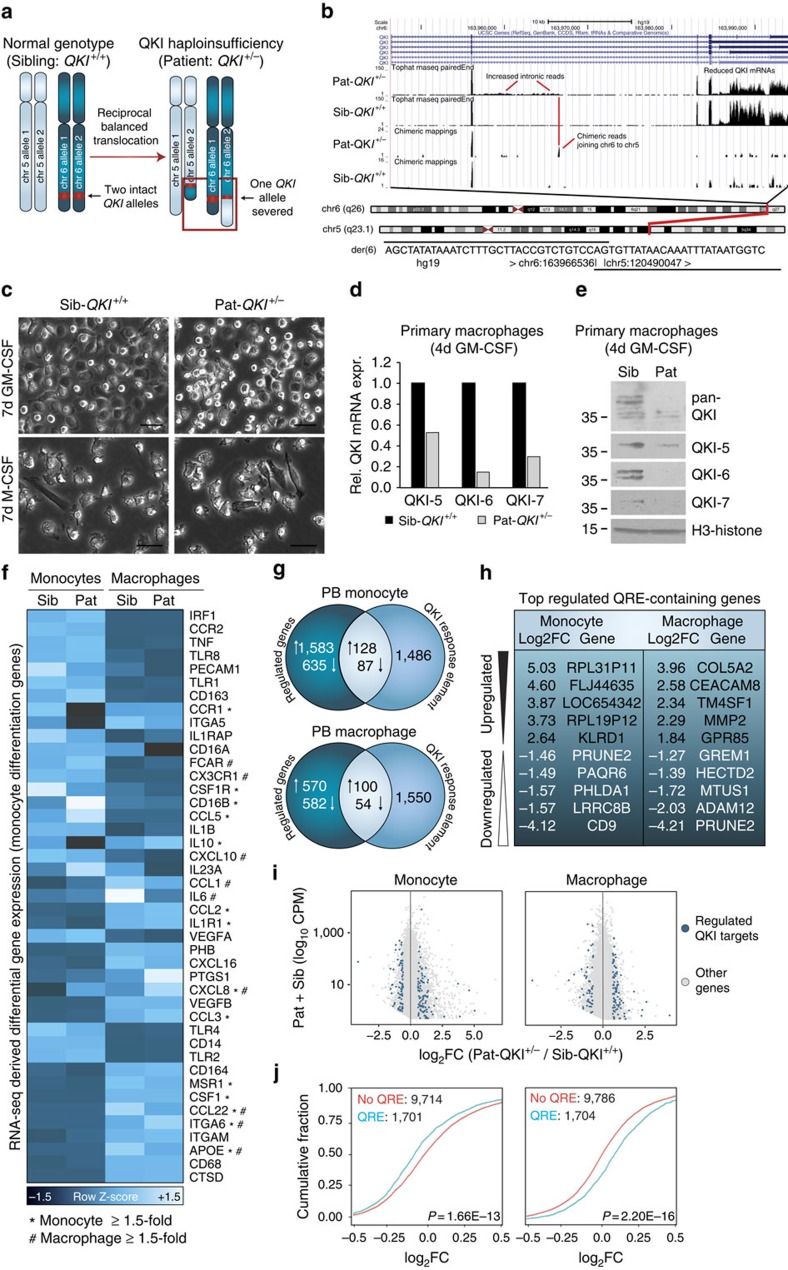
Characterization of monocyte and macrophage biology in a unique QKI haploinsufficient patient. (**a**) Schematic of chromosomal translocation event in the *qkI* haploinsufficient patient (Pat-*QKI*^*+/−*^), reducing QKI expression to ∼50% that of her sister control (Sib-*QKI*^*+/+*^). (**b**) Top: UCSC Genome Browser display of reference genome QKI locus with standard and chimeric reads for the patient and sibling. The reduced expression levels and altered 3′-untranslated region (UTR) composition in the patient RNA as compared with a sibling control is noteworthy. Patient shows increased intronic RNA extending to the point where chimeric reads map at the breakpoint to chr5. Middle: chromosome diagrams showing normal chromosomes 5 and 6 with the red line, indicating the location of the breakage fusion point. Bottom: sequence across the fusion point. The chromosomal origin of the AG dinucleotide is ambiguous. (**c**) Photomicrographs of sibling and patient macrophages, cultured in GM-CSF or M-CSF for 7 days, respectively. (**d**,**e**) Assessment of QKI isoform mRNA and protein expression in 4-day GM-CSF-stimulated macrophages in the sibling and patient. (**f**) Hierarchical clustering (Euclidean algorithm) of key monocyte differentiation genes depicting changes in RNA-seq-derived mRNA abundance where dark blue=low expression, whereas light blue=high expression (* and/or # indicates ≥1.5-fold expression change in monocytes or macrophages, respectively). (**g**) Venn diagrams with numbers of differentially expressed genes (minimally ±1.5-fold; patient/sibling expression) for unstimulated (top) and GM-CSF stimulated macrophages (bottom). An expression cutoff (Pat+Sib expression≥1CPM) was applied. (**h**) The most differentially expressed genes, harbouring a QRE are depicted. (**i**) Genome-wide scatterplot of mRNA abundance (*y* axis: Log_10_ CPM) versus the log_2_FC (*x* axis: Patient/sibling CPM) after an expression cutoff (Pat+Sib expression ≥1 CPM) in monocytes (left) and GM-CSF-stimulated macrophages (right). Blue dots indicate QRE-containing transcripts minimally ±1.5-fold differentially expressed. Grey dots do not fulfill these criteria. (**j**) CDF (*y* axis) for QKI target (QRE containing: blue line) and non-target (non-QRE containing: cyan line) mRNAs (*x* axis: log_2_FC) in monocytes (left) and macrophages (right). Left shift indicates lower expression of QKI target genes, whereas a right shift indicates higher expression of QKI targets in the patient samples. Distributions were compared using a Wilcoxon rank-sum test.

To further assess the role of QKI in monocyte and macrophage biology, we undertook an in-depth analysis of a unique, QKI haploinsufficient individual (Pat-*QKI*^*+/−*^) and her sister control (Sib-*QKI*^*+/+*^)^38^. This patient is the only known carrier of a balanced reciprocal translocation (t(5;6)(q23.1;q26)), where a breakpoint in one of her *QKI* alleles specifically reduces QKI expression by 50% in both QKI mRNA^38^ and QKI protein levels as compared with her sibling (Sib-*QKI*^*+/+*^; Fig. 3a,b). RNA sequencing (RNA-seq) analysis (see below) confirmed altered QKI expression and furthermore revealed the precise location of the translocation breakpoint in intron 4 of QKI (Fig. 3b and Supplementary Fig. 1a).

We next compared the circulating monocytes of these two individuals for the expression of well-established monocyte cell surface markers such as CD14, CD16, CX3CR1, CCR2, SELPLG and CSF1R by fluorescence-activated cell sorting (FACS) analysis. Although monocyte subset ratios were not different (Supplementary Fig. 2a), the expression of CSF1R, the receptor that drives macrophage commitment, was elevated in Pat-*QKI*^*+/−*^ non-classical monocytes as compared with Sib-*QKI*^*+/+*^ (Supplementary Fig. 2b). As CSF1R is normally reduced when monocytes differentiate into macrophages, this observation points towards a potential defect in monocyte maturation in the patient.

Next, we investigated the consequences of decreased QKI expression on monocyte to macrophage differentiation. For this, we obtained freshly isolated Pat-*QKI*^*+/−*^ and Sib-*QKI*^*+/+*^ monocytes from venous blood and treated the cells for 7 days in the presence of either GM-CSF or M-CSF. Similar to the results in Fig. 2b, Sib-*QKI*^*+/+*^ monocytes possessed the capacity to adopt the characteristic pro-inflammatory macrophage morphology, whereas monocytes from Pat-*QKI*^*+/−*^ generally retained a monocytic morphology (Fig. 3c top panels). We harvested RNA and protein from these macrophages and found that both QKI mRNA and protein levels were decreased (Fig. 3d,e). Surprisingly, this reduction in QKI did not appear to have an impact on the conversion of monocytes into anti-inflammatory macrophages (Fig. 3c bottom panels), a finding that prompted us to focus on the role of QKI in monocyte to macrophage differentiation in a pro-inflammatory setting.

### QKI impacts transcript abundance in monocytes and macrophages

The observed increase in QKI expression during macrophage differentiation and well-established function as a splicing and translational regulator^23^,^31^,^39^,^40^ suggested that QKI is necessary for posttranscriptional control of events that lead to macrophage identity. To identify potential regulatory targets of QKI at a genome-wide level, we characterized the transcriptomes of Sib-*QKI*^*+/+*^ and Pat-*QKI*^*+/−*^ monocytes and GM-CSF-stimulated macrophages by RNA-seq (Supplementary Data 2). First, we assessed the expression levels of established immune-regulated genes^11^,^12^. As shown in Fig. 3f, the mRNA levels of many monocyte to macrophage differentiation markers^11^,^12^ were similarly regulated in Sib-*QKI*^*+/+*^ and Pat-*QKI*^*+/−*^ (*CD68↑, ApoE↑, ITGAM↑, CD14↓, CX3CR1↓* and *CD163↓*). In contrast, the expression levels of several key pro- and anti-inflammatory markers indicated an anti-atherogenic shift in Pat-*QKI*^*+/−*^ macrophages (Fig. 3f right; *IL6↓, IL23a↓, CD16A↓, CD16B↓, ApoE↓* and *IL10↑*). At the genome-wide level, QKI haploinsufficiency altered the abundance of 2,433 and 1,306 mRNA species in monocytes and macrophages (Fig. 3g, Supplementary Data 2 and Supplementary Fig. 3 top), respectively. Subsequently, we computationally determined the subset of mature mRNA transcripts in the genome that contain a QKI-binding sequence motif (termed QRE)^30^ (Supplementary Data 2). Our data suggested that QKI directly modulates the expression of 215 (128↑ and 87↓) and 154 (100↑ and 54↓) mRNAs in PB monocytes and macrophages, respectively (Fig. 3g, Venn sum of intersect). The five most differentially expressed genes in the patient relative to the sibling that harbour a QRE are shown in Fig. 3h. By selecting genes containing QREs, we identified a substantial number of putative QKI-mediated changes in transcript abundance (Fig. 3i).

Previous genome-wide studies have reported contrasting roles for QKI as both a repressor and stabilizer of target mRNAs^31^,^33^. Intrigued by this ambiguity, we determined the consequences of QKI haploinsufficiency on mRNA transcript abundance in monocytes and macrophages. For this, we tested whether the presence of a QRE within a target mRNA was associated with increased or decreased mRNA abundance in the patient relative to her sibling (Supplementary Fig. 3 top). For this, we plotted the cumulative distribution fraction (CDF: *y* axis, as a fraction of total genes) against the transcript Log_2_FC (*x* axis: Pat-*QKI*^*+/−*^/Sib-*QKI*^*+/+*^) and stratified for either putative QKI targets (with QRE) or non-targets (no QRE). In PB monocytes, a reduction in QKI was associated with significantly lowered target mRNA expression (Fig. 3j left panel, left shift of cyan line). In contrast, in PB macrophages the expression levels of mature mRNAs containing QREs was strikingly increased in the patient relative to her sibling, as compared with those without QREs (Fig. 3j right panel, right shift of cyan line). Collectively, these studies suggested that QKI potently regulates gene expression during monocyte-to-macrophage differentiation.

### QKI controls splicing in monocytes and macrophages

**Figure 4:**
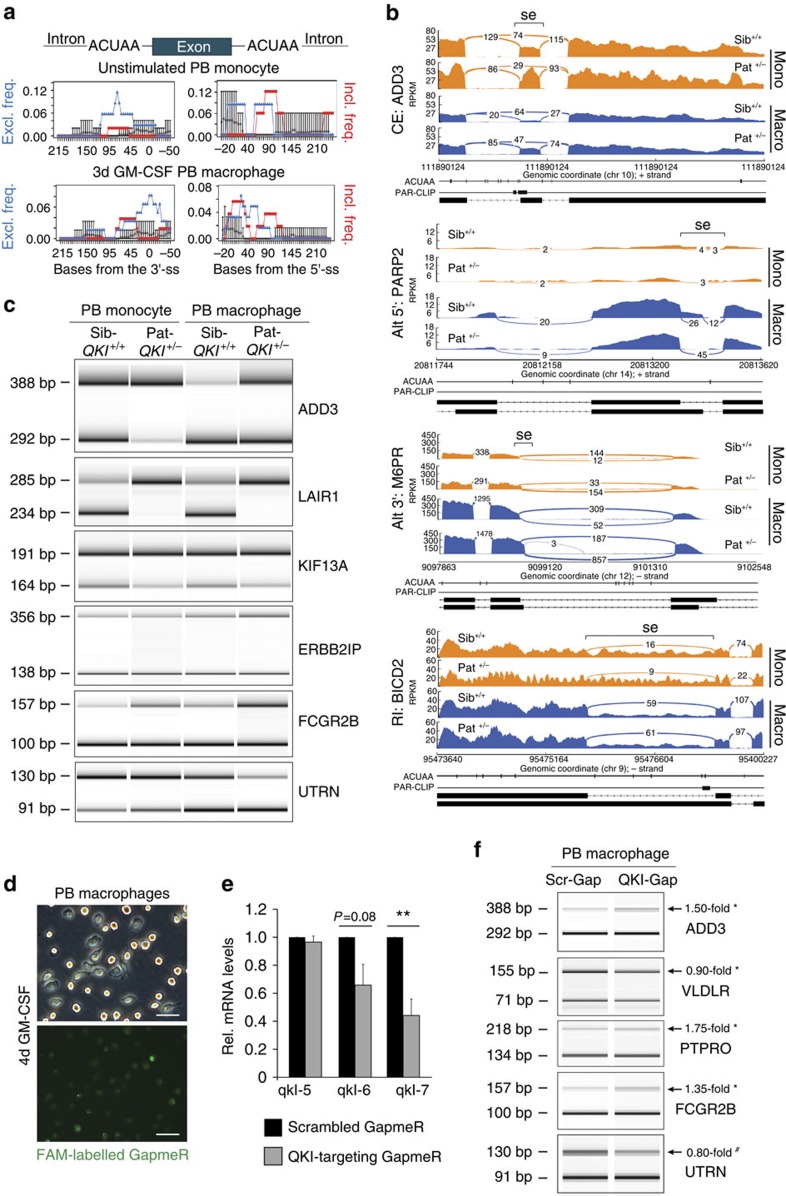
QKI influences pre-mRNA splicing in naive PB monocytes and macrophages. (**a**) SpliceTrap assessment of the proximal ACUAA RNA motif enrichment in 50-bp windows upstream and downstream of alternatively spliced cassette exons (as compared with a background set of exons; grey circles). The relationship between the frequency of exon exclusion (blue triangles) or exon inclusion (red squares) and ACUAA RNA motif enrichment over the genomic locus are depicted. (**b**) Sashimi plots illustrate RNA-seq read coverage for selected alternative splicing events in Pat-*QKI*^*+/−*^ versus Sib-*QKI*^*+/+*^ PB monocytes (orange) and macrophages (blue). Splicing events (se) are highlighted by inverted brackets. The location of ACUAA motifs and QKI PAR-CLIPs are provided below. Splicing events were defined based on the genomic organization of RefSeq transcripts (bottom tracks). Full event details are provided in Supplementary Data 3. (**c**) PCR validation of alternatively spliced cassette exons in Sib-*QKI*^+/+^ and Pat-*QKI*^+/*−*^ PB-derived monocytes and macrophages. Primers were designed to target constitutive flanking exons. PCR product size for exon inclusion (top) and exclusion (bottom) variants are provided (left). (**d**) Phase-contrast and fluorescence-microscopy photographs (scale bar, 50 μm) of primary human, PB macrophages of healthy controls that have been treated with FAM-labelled GapmeRs, to reduce QKI expression. (**e**) Quantitative RT–PCR (qRT–PCR) of QKI mRNA isoform expression in GapmeR-treated macrophages (*n*=3). Data expressed as mean±s.e.m.; Student's *t*-test, with **P<0.01. (**f**) PCR validation of alternatively spliced cassette exons in GapmeR-treated PB-derived macrophages. Primers were designed to target constitutive flanking exons. PCR product size for exon inclusion (top) and exclusion (bottom) variants are provided (left). A representative illustration is shown of an *n*=3 donors. Data expressed as mean±s.e.m.; Student's *t*-test, with **P<0.01 and #*P*=0.08.

Given previous reports that QKI is involved in splicing of pre-mRNAs^23^,^39^,^40^,^41^,^42^, we tested whether QKI acts similarly in monocytes and macrophages. First, we evaluated our RNA-seq analysis of Sib-*QKI*^*+/+*^ and Pat-*QKI*^*+/−*^ PB monocytes and macrophages for splicing changes (Supplementary Data 3). This analysis uncovered 1,513 alternative splicing events between Pat-*QKI*^*+/−*^ and Sib-*QKI*^*+/+*^ monocytes and macrophages, revealing events that were unique to either monocytes or macrophages, as well as common events (Supplementary Data 3). Previous observations for QKI and other RBPs suggested that when a splicing factor binds the intron downstream of an alternative exon, it promotes exon inclusion; however, when binding the intron upstream of the alternative exon, the RBP promotes exon skipping^19^,^43^. We analysed the RNA-seq data for such a trend using the set of splicing events that change between the Pat-*QKI*^*+/−*^ and Sib-*QKI*^*+/+*^, to determine the frequency of the QKI-binding motif, ACUAA, around these regulated exons, relative to a background set of exons that is expressed, but inclusion is unchanged between the two data sets. The results of these analyses are shown in Fig. 4a and Supplementary Data 4, and demonstrate ACUAA motif enrichment upstream of exons with increasing inclusion in Pat-*QKI*^*+/−*^ (QKI repressed exons) relative to background exons, as well as an increase in ACUAA motif frequency downstream of exons with increased skipping (QKI activated) relative to background. This suggested that similar to C2C12 myoblasts^39^, QKI promotes exon skipping by binding the upstream intron, while promoting inclusion of alternative exons by binding to the downstream intron, in monocytes and in macrophages. These data strongly support a direct, position-dependent role for QKI in regulating alternative splicing, while also providing additional protein diversity during monocyte-to-macrophage differentiation.

As shown in Fig. 4b, QKI haploinsufficiency triggered alternative splicing events in PB monocytes (orange tracks) and macrophages (blue tracks). Interestingly, the presence of QKI-binding sites, as defined by either experimentally determined QKI PAR-CLIP sites^39^ and/or ACUAA motifs clearly associated with changes in exon inclusion (for example, *ADD3*), alternative 5′-splice sites (PARP2), alternative 3′-splice sites (M6PR) and intron retention (for example, BICD2), thereby expanding the detection of veritable QKI-regulated events beyond cassette exons (splice event ‘se' location defined by brackets). Importantly, strong correlations were observed between QKI expression levels and the magnitude of the splicing event, be it between the patient and sibling control, or between monocytes and macrophages (Fig. 4b).

Finally, we validated several alternatively spliced cassette exons, including events in ADD3, LAIR1 and UTRN by reverse transcriptase–PCR (RT–PCR), using primers in flanking exons (Fig. 4c). Collectively, our RNA-seq data analysis of this unique QKI haploinsufficient individual strongly suggested a direct role for QKI in regulating alternative splicing events that could influence monocyte to macrophage differentiation.

To extend results obtained with the QKI haploinsufficient patient, we abrogated QKI expression in naive primary human monocytes harvested from freshly drawn venous blood of healthy controls. We designed GapmeR antisense oligonucleotides that either targeted QKI for degradation (QKI-Gap), or are scrambled as a control (Scr-Gap), coupled with a 5′-FAM label to track their cellular uptake. The QKI-Gap and Scr-Gap compounds were administered to the freshly isolated monocytes, concomitant with GM-CSF for 96 h, to drive the differentiation to pro-inflammatory macrophages. In contrast to our attempts to reduce QKI mRNA levels using other well-established approaches, we observed virtually no signs of cytotoxicity or apoptosis following GapmeR treatment. Furthermore, the treatment did not hamper the capacity of monocytes to differentiate into macrophages (Fig. 4d top), while uptake efficiency approached 100% (based on FAM^+^ cells; Fig. 4d bottom). After 96 h, we harvested RNA from the QKI-Gap- and Scr-Gap-treated macrophages, which yielded a minimal reduction in QKI-5 mRNA levels but remarkable reductions in QKI-6 and QKI-7 mRNAs (Fig. 4e). Albeit that the GapmeR-mediated reduction in QKI expression in primary human macrophages was not as striking as that observed in the QKI haploinsufficient patient, it nonetheless enabled us to visualize and validate significant changes in several of the aforementioned QKI-mediated alternative splicing events, such as *ADD3* and FcγR-IIb (*FCGR2B*) (Fig. 4f; *n*=3 donors). It should be noted that the inability to remarkably reduce the expression of the nuclear QKI isoform, namely QKI-5, could be responsible for the discrepancy between the striking shift in splicing observed in the QKI haploinsufficinet patient as compared with the GapmeR-mediated abrogation of QKI expression. Taken together, these studies clearly pinpoint QKI as a regulator of pre-mRNA splicing during monocyte-to-macrophage differentiation and implicate QKI gene dosage as a determinant of splicing event magnitude.

### QKI regulates transcript abundance in THP-1 cells

**Figure 5:**
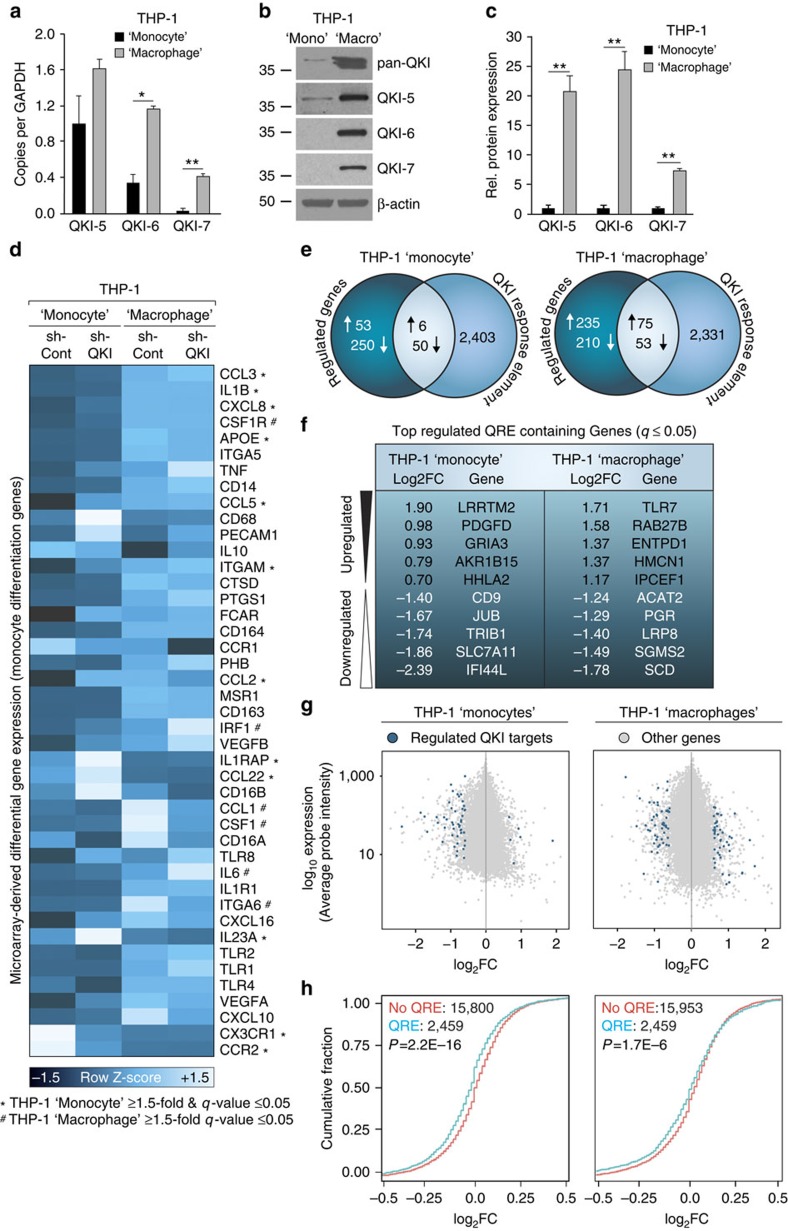
QKI influences mRNA transcript abundance during differentiation of THP-1 monocyte-like cells to THP-1 macrophage-like cells. (**a**) mRNA expression of the QKI isoforms as compared with glyceraldehyde 3-phosphate dehydrogenase (GAPDH) in THP-1 ‘monocytes' and 8 days differentiated THP-1 ‘macrophages' (biological *n*=3). Data expressed as mean±s.e.m.; Student's *t*-test; *P<0.05 and **P<0.01. (**b**) Western blot analysis of whole-cell lysates of THP-1 ‘monocytes' and THP-1 ‘macrophages'. (**c**) Western blot quantification of QKI protein isoforms, normalized to β-actin in THP-1 ‘monocytes' and THP-1 ‘macrophages' (*n*=3). Data expressed as mean±s.e.m.; Student's *t*-test; **P<0.01. (**d**) Hierarchical clustering (Euclidean algorithm) of key monocyte differentiation genes depicting changes in microarray-derived mRNA abundance THP-1 ‘monocytes' (left two lanes) and THP-1 ‘macrophages' (right two lanes), where dark blue=low expression, whereas light blue=high expression (* and/or # beside gene name is indicative of a significant ≥1.5-fold change in expression in monocytes or macrophages, respectively). (**e**) Venn diagrams depicting the number of microarray-derived differentially expressed genes (minimally ±1.5-fold; sh-QKI/sh-Cont expression, *q*-value≤0.05) for unstimulated THP-1 ‘monocytes' (left Venn diagram) and THP-1 ‘macrophages' (right Venn diagram). (**f**) The most significantly differentially expressed genes harbouring a QRE are shown. (**g**) Genome-wide scatterplot of mRNA abundance in THP-1 ‘monocytes' (left scatterplot) and THP-1 ‘macrophages' (right scatterplot); *y* axis: Log_10_ probe intensity versus the *x* axis: log2FC: sh-QKI average probe intensity/sh-Cont average probe intensity. Blue dots indicate QRE-containing transcripts that are minimally ±1.5 fold differentially expressed (*q*≤0.05). Grey dots do not fulfill these criteria. (**h**) CDF (*y* axis) for QKI target (QRE containing: blue line) and non-target (non-QRE containing: cyan line) mRNAs (*x* axis: log_2_FC) in THP-1 ‘monocytes' (left plot) and THP-1 ‘macrophages' (right plot). Left shift indicates lower expression of QKI target genes in the sh-QKI samples, whereas a right shift is indicative of higher expression of QKI targets in the sh-QKI samples. Distributions were compared using a Wilcoxon rank-sum test.

To provide further support for a regulatory role for QKI during monocyte-to-macrophage differentiation, we tested whether QKI could similarly modulate transcript abundance and pre-mRNA splicing in a well-established monocyte cell line, namely THP-1 cells. Similar to GM-CSF-induced differentiation of PB monocytes into macrophages, the 12,13-phorbol myristyl acetate (PMA)-induced transition of THP-1 ‘monocytes' to ‘macrophages' was associated with the following: (1) significantly increased expression of all QKI mRNA transcripts (Fig. 5a); (2) barely detectable levels of QKI protein in THP-1 ‘monocytes' (Fig. 5b and Supplementary Fig. 4a); and (3) significantly increased expression of QKI protein during THP-1 ‘monocyte' to ‘macrophage' differentiation (Fig. 5b,c). Next, we stably transduced THP-1 ‘monocytes' with either short-hairpin RNA (shRNA) targeting QKI (sh-QKI) to specifically deplete QKI or with a non-targeting shRNA control (sh-Cont) (Supplementary Fig. 4b). Similar to GM-CSF-stimulated Pat-*QKI*^*+/−*^ versus Sib-*QKI*^*+/+*^ monocytes, sh-QKI THP-1 ‘monocytes' displayed an inability to adopt the ‘macrophage' morphology following stimulation with PMA as compared with sh-Cont THP-1 ‘monocytes' (Supplementary Fig. 4c arrows). We subsequently determined mRNA levels using an exon junction microarray^44^ analysing RNA isolated from unstimulated and 3 days PMA-stimulated THP-1 sh-Cont and sh-QKI 'monocytes' and ‘macrophages' (Supplementary Data 5). Next, as shown in Fig. 5d, we assessed the expression profile of established monocyte differentiation genes (for example, *CD14↑, CXCL8↑, CSF1R↑, ApoE↑, CX3CR1↓, CCR2↓* and *CCL22↓*). Similar to QKI haploinsufficient macrophages, several markers in sh-QKI THP-1 ‘macrophages' displayed an anti-atherogenic phenotypic shift (*IL6↓, IL23a↓, CD16A↓, CD16B↓, ApoE↓ and IL10↑*) as compared with sh-Cont THP-1 ‘macrophages' (Fig. 5d).

At the genome-wide level, the reduction of QKI significantly altered the abundance of 359 and 573 mRNAs in THP-1 ‘monocytes' and ‘macrophages', respectively (Fig. 5e, Supplementary Data 5 and Supplementary Fig. 3 bottom). Of these differentially expressed mRNAs, 56 and 128 were computationally predicted QKI targets based on the presence of a QRE in the mature mRNA (Fig. 5e intersect). The most differentially expressed transcripts harbouring a QRE are denoted in Fig. 5f. The expression levels of mRNAs targeted by QKI in THP-1 ‘monocytes' and ‘macrophages' are depicted in Fig. 5g (blue dots) and Fig. 5h (blue lines), relative to those not directly affected by changes in QKI levels (Fig. 5g grey dots and Fig. 5h cyan lines). Consistent with our analyses in PB monocytes, putative direct QKI target mRNAs were mostly reduced on a targeted QKI reduction in THP-1 ‘monocytes' (Fig. 5h, left plot), although a shift towards increased target mRNA abundance in THP-1 ‘macrophages' was not observed (Fig. 5h, right plot).

### QKI modifies pre-mRNA splicing patterns in THP-1 cells

**Figure 6:**
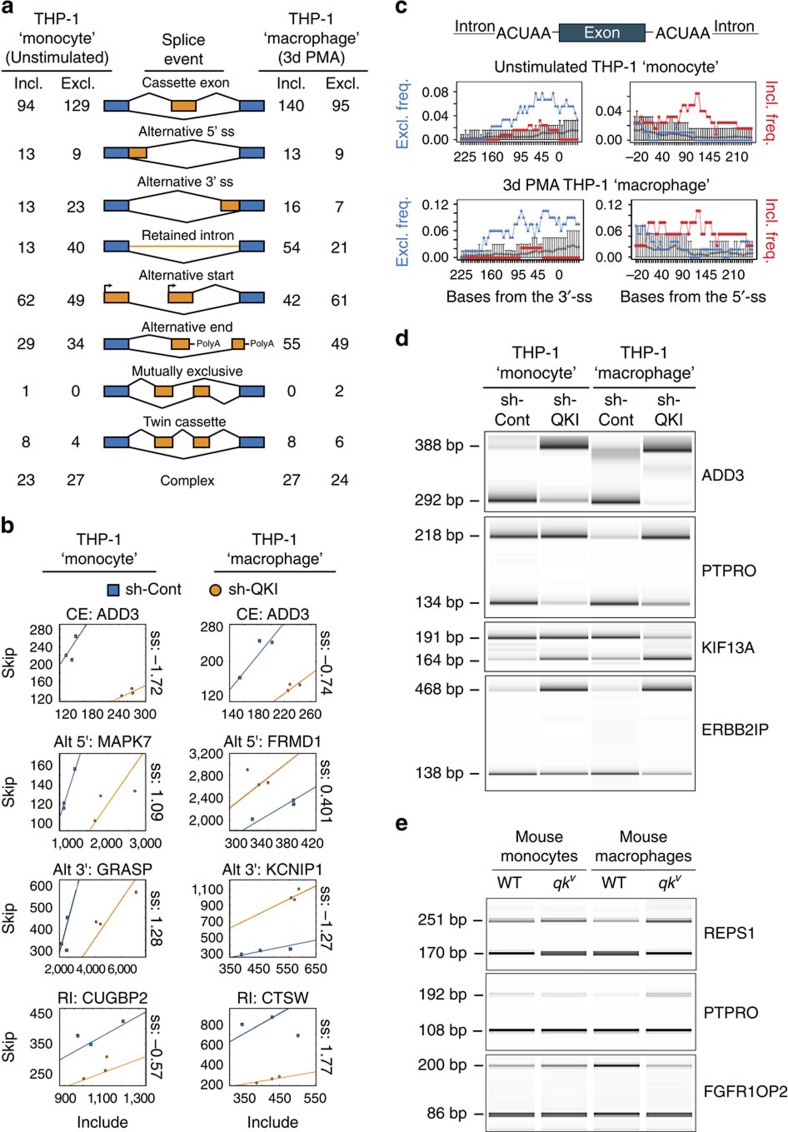
QKI expression levels influence pre-mRNA splicing during THP-1-based monocyte-like to macrophage-like cell differentiation. (**a**) Schematic depicting detectable alternative splicing events with the splicing-sensitive microarray platform and number of inclusion (incl.; top lines) or exclusion (excl.; bottom lines) events observed in unstimulated THP-1 ‘monocytes' (left) and 3-day PMA-stimulated THP-1 ‘macrophages' (*n*=3, *q*≤0.05). (**b**) Scatterplots of skip (*y* axis) and include (*x* axis) probe set intensity for selected alternative splicing events in sh-Cont (blue boxes) versus sh-QKI (orange circles) in unstimulated and 3 days PMA-stimulated THP-1 ‘monocytes' and ‘macrophages', respectively. Regression coefficients (constrained to pass the origin) are depicted as solid lines. The log_2_ difference in the slopes (termed separation score; ss) are provided to the right of the plots for each event, with for example, an ss of −1.72, indicating a 3.3-fold more inclusion of ADD3 exon 13 in sh-QKI versus sh-Cont THP-1 ‘monocytes'. Full event details are provided in Supplementary Data 6. CE, cassette exon; Alt 5′ or 3′, alternative 5′ or 3′ splice site; RI, retained intron. (**c**) SpliceTrap assessment of average proximal ACUAA RNA motif enrichment in 50 bp windows upstream and downstream of alternatively spliced cassette exons as compared with a background set of exons (grey circles). The relationship between the frequency of exon exclusion (blue triangles) or exon inclusion (red squares) and ACUAA RNA motif enrichment are depicted. (**d**) PCR validation of alternatively spliced cassette exons in sh-Cont and sh-QKI THP-1 ‘monocytes' and ‘macrophages'. Primers were designed to target constitutive flanking exons. PCR product size for exon inclusion (top) and exclusion (bottom) variants are provided (left). All experiments depict biological *n*=3. (**e**) PCR validation of three splicing events in *wt* and *qk*^*v*^ mouse-derived primary monocytes and 7 days M-CSF-stimulated macrophages. PCR product size for exon inclusion (top) and exclusion (bottom) variants are provided (left). Depicted is a representative PCR for at least a biological *n*≥3.

Having identified that QKI haploinsufficiency generates pre-mRNA splicing events that probably have an impact on monocyte and macrophage biology, we also analysed RNA isolated from sh-Cont and sh-QKI THP-1 ‘monocytes' and ‘macrophages' for alternative splicing events using the exon junction microarray platform^44^. This highly sensitive technology uses probes that are designed specifically to detect both constitutive exon–exon junctions and alternative exon–exon junctions, enabling one to quantify inclusion ratios for alternative splicing events. These studies uncovered 571 and 629 differentially regulated alternative splicing events in THP-1 ‘monocytes' and ‘macrophages', respectively, including numerous cassette exons, alternative 5′- and 3′-splice sites, and retained introns (Fig. 6a and Supplementary Data 6; *n*=3). Detected splicing events are illustrated in Fig. 6b, where the skip and include intensities (*y* axis and *x* axis, respectively) of transcript-specific hybridization probes directed to either the constitutive or alternatively spliced exons are plotted. The separation score, obtained by determining slope differences, indicates the magnitude of the splicing event. Similar to the motif enrichment analyses performed for the RNA-seq of PB monocytes and macrophages, these studies confirmed that exon skipping frequency was significantly correlated with alternative exons that had an ACUAA motif in the upstream intron (Fig. 6c left panels and Supplementary Data 4). In contrast to the subtle enrichment of inclusion frequency observed in Pat-*QKI*^*+/−*^ and Sib-*QKI*^*+/+*^ monocytes and macrophages (Fig. 3a right panels), exon inclusion frequency in THP-1 ‘monocytes' and ‘macrophages' was clearly associated with the presence of ACUAA motifs in the downstream intron (Fig. 6c and Supplementary Data 4).

Finally, alternative cassette exons in THP-1 ‘monocytes' and ‘macrophages' were PCR validated (Fig. 6d). Importantly, we also selected several top splicing events from THP-1 ‘monocytes' and ‘macrophages' (Supplementary Data 6), and validated these in RNA harvested from *wt* and *qk*^*v*^ mice, including REPS1, PTPRO and FGFR1OP2 (Fig. 6e).

### QKI targets monocyte activation and differentiation pathways

We subsequently determined how QKI-induced changes in mRNA transcript abundance could have an impact on Gene Ontology (GO) enrichment of coordinately regulated pathways during monocyte-to-macrophage differentiation. As shown in Table 1 and Supplementary Data 7, these GO analyses point towards a central regulatory role for QKI in immune responses to injury, processes that play a critical role in the onset and development of atherosclerosis and other inflammation-based diseases. In both monocytes and macrophages, changes in QKI expression clearly had an impact on Liver X Receptor (LXR)/Retinoid X Receptor (RXR) activation and Peroxisome Proliferator-Activated Receptor (PPAR) activation and signalling, implicating a key role for QKI in regulating cholesterol biosynthesis and metabolism. Furthermore, a reduction in QKI expression also appeared to influence T-cell and Toll-like receptor signalling, biological processes that play prominent roles in the rapid resolution of infection, while in chronic settings exacerbate inflammatory conditions. Finally, the gene enrichment analysis suggested that posttranscriptional processing of factors driving the recruitment, adhesion and diapedesis of immune cells were affected by changes in QKI expression.

### QKI facilitates monocyte adhesion and migration

**Figure 7:**
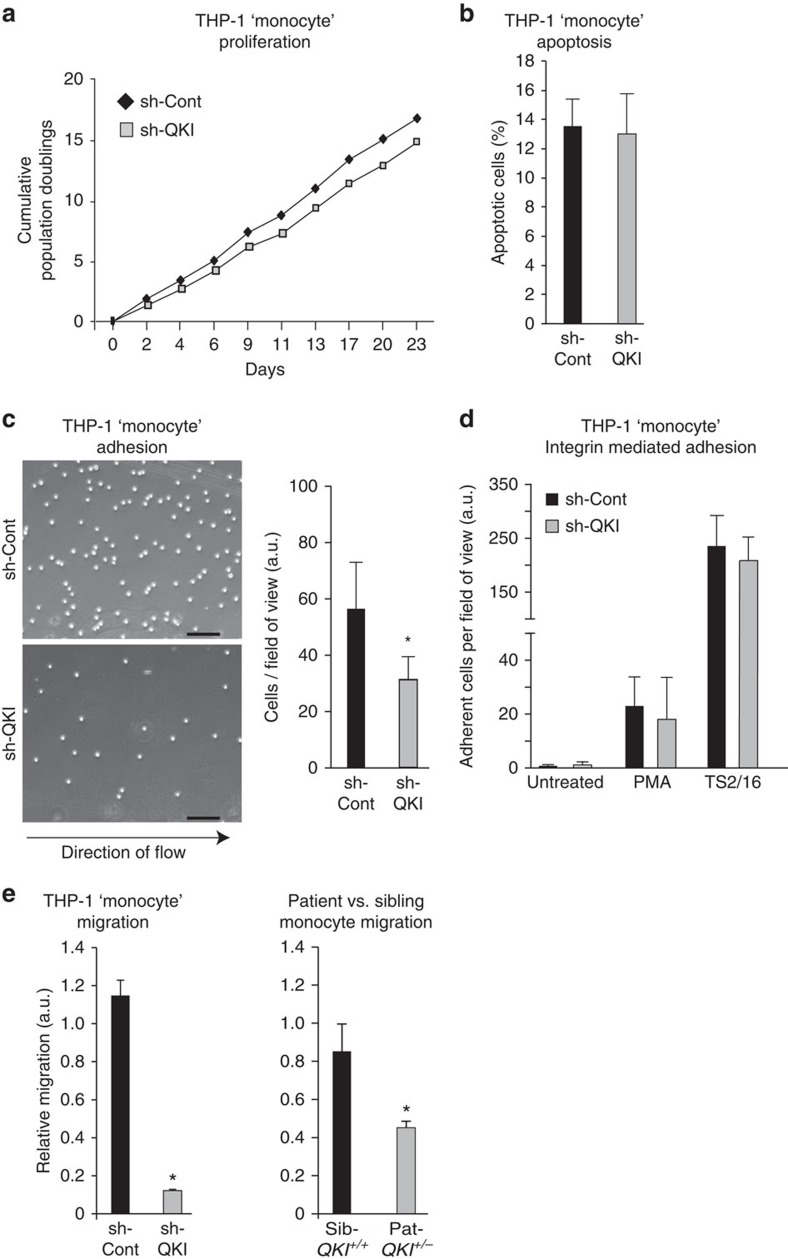
QKI expression levels have an impact on monocyte adhesion as well as migration and differentiation. (**a**) Cumulative population doublings (*y* axis: CPDs) were counted to assess the effect of QKI reduction on cellular proliferation over time (*x* axis: days). Population growth curves were compared using linear regression analysis.(**b**) Quantification of cellular apoptosis, where annexin V^+^ and propidium iodide^+^ cells were categorized as apoptotic, as determined by FACS analysis. (**c**) Quantification of sh-Cont and sh-QKI THP-1 ‘monocyte' adhesion to collagen matrix pretreated with platelet-rich plasma under flow, mimicking *in-vivo* endothelial denudation. Direction of flow is indicated below the photomicrographs (*n*=3). Data expressed as mean±s.e.m.; Student's *t*-test; *P<0.05. Scale bar, 100 μm. (Also see Supplementary Movies 1 and 2). (**d**) Assessment of integrin-mediated adhesion. Quantification of adhesion to collagen for untreated, PMA- or TS2/16-treated sh-Cont and sh-QKI THP-1 ‘monocytes' are plotted. TS2/16 is an antibody that turns all β1-integrins in the high-affinity conformation, inducing cellular adhesion. (**e**) Quantification of cellular transwell migration towards either fMLP (for THP-1 ‘monocytes') or macrophage chemoattractant protein 1 (MCP-1; for PB monocytes from either sibling or patient (*n*=4 technical replicates). Data expressed as mean±s.e.m.; Student's *t*-test; *P<0.05 and **P<0.01.

Our experimentally determined changes in (pre)-mRNA splicing and expression, as well as bioinformatically predicted changes in biological processes, prompted us to evaluate whether these QKI-induced posttranscriptional modifications could affect monocyte and macrophage function. To test this, we first assessed whether cell survival is affected by a reduction of QKI expression in THP-1 ‘monocytes'. Importantly, the cumulative population doublings and apoptotic rates were not affected by decreased QKI levels (Fig. 7a,b). Next, we assessed cell adhesion to glass coverslips treated with effector molecules (collagen and activated platelets) in the presence of fluid shear stress, an experimental design that mimics the response of monocytes to endothelial denudation in the vessel^45^. Live-cell imaging clearly showed that the shRNA-mediated depletion of QKI in THP-1 ‘monocytes' reduced cellular adhesion under flow conditions, as evidenced by their continued rolling along the substrate and inability to firmly attach (Fig. 7c and Supplementary Movies 1 and 2). This firm adhesion of monocytes is aided by the activation of β1-integrins on the cell surface that mediate high-affinity interactions with the extracellular matrix at sites of injury^36^. We tested whether QKI depletion had an impact on β1-integrin function by incubating sh-Cont and sh-QKI THP-1 ‘monocytes' with an antibody (TS2/16) that forces β1-integrins into the activated, adhesive conformation^37^. Interestingly, the abrogation of QKI did not affect monocyte adhesion properties in this setting (Fig. 7d), indicating that proper integrin expression and functionality is not dependent on QKI.

We subsequently tested whether QKI expression levels could have an impact on monocyte migration *in vitro* by seeding sh-QKI or sh-Cont THP-1 ‘monocytes' into transwell migration chambers and assessed their ability to migrate towards the chemoattractant formyl-methionyl-leucyl-phenylalanine (fMLP). Indeed, depletion of QKI in monocytes inhibited migration (Fig. 7e). This finding prompted us to similarly assess the capacity of Pat-*QKI*^+/*−*^ and Sib-*QKI*^+/+^ monocytes freshly isolated from venous blood to migrate to macrophage chemoattractant protein 1, a physiologic recruiter of monocytes at sites of vascular injury. These studies revealed a significant reduction in transwell migration for Pat-*QKI*^+/*−*^ monocytes (Fig. 7e), validating our findings in THP-1 ‘monocytes', and provided evidence that QKI influences monocyte adhesion and migration in inflammatory settings.

### QKI drives foam cell formation

**Figure 8:**
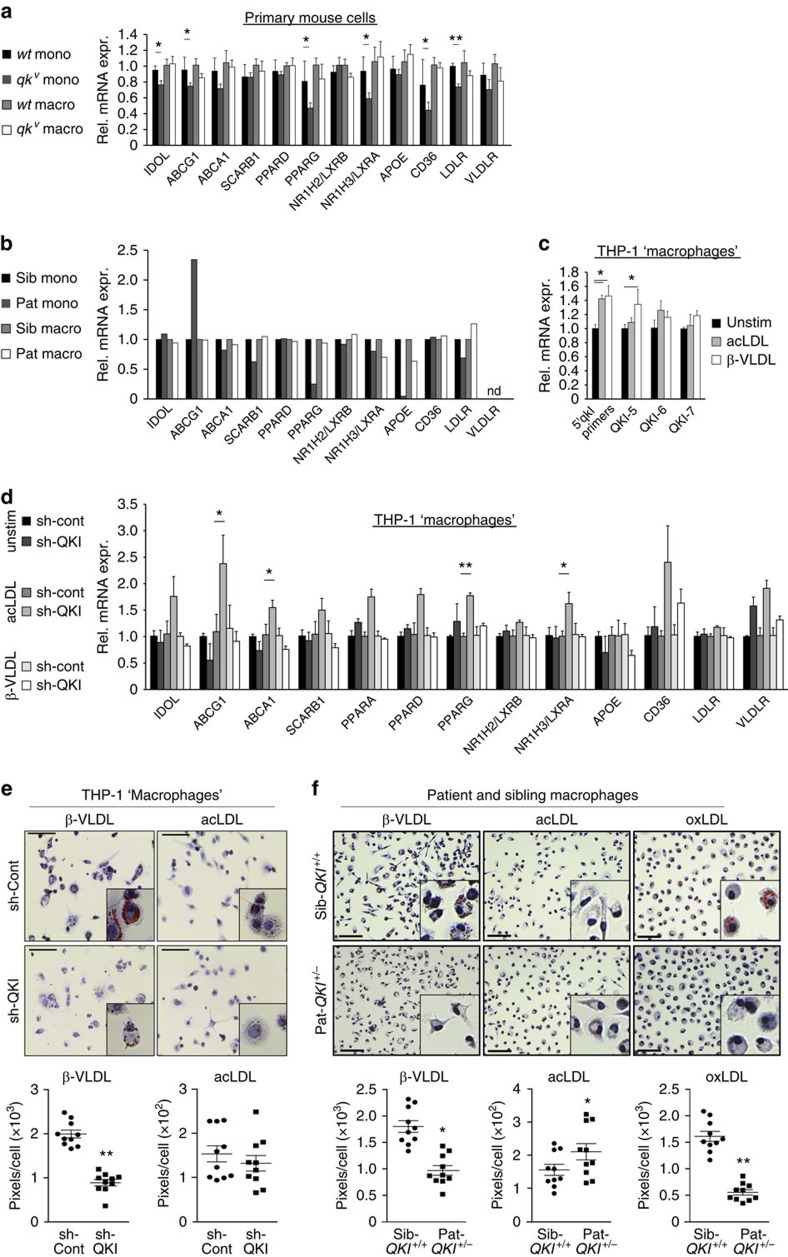
QKI regulates the expression of atherosclerosis-related mRNAs and impairs foam cell formation. (**a**) Quantitative RT–PCR (qRT–PCR) analysis of established atherosclerosis-related genes in *wt* of *qk*^*v*^-derived monocytes and macrophages. Gene expression in *qk*^*v*^ samples are relative to either *WT* monocytes or *WT* macrophages (*n*≥3). Data expressed as mean±s.e.m.; Student's *t*-test; *P<0.05, **P<0.01. (**b**) RNA-seq-derived expression values to illustrate the expression of established atherosclerosis-related genes in sibling or patient monocytes and macrophages. (**c**) qRT–PCR analysis of QKI isoform mRNA expression in unstimulated THP-1 ‘macrophages', or treated with β-VLDL or acLDL for 24 h. Data expressed as mean±s.e.m.; one-way analysis of variance (ANOVA), Bonferroni's *post-hoc* test; *P<0.05. (**d**) qRT–PCR analysis of well-known atherosclerosis-related genes in sh-cont or sh-QKI THP-1 ‘macrophages' that were either left untreated or treated with acLDL or β-VLDL to induce foam cell formation (*n*=3). Data expressed as mean±s.e.m.; Student's *t*-test; *P<0.05 and **P<0.01. (**e**) Photomicrographs of an Oil-red-O staining to assess β-VLDL, acLDL uptake in either sh-Cont or sh-QKI THP-1 ‘macrophages'. Scale bar, 100 μm. Inset is a high-magnification image of lipid-droplet accumulation. Data expressed as mean±s.e.m.; Student's *t*-test; **P<0.01. (**f**) Photomicrographs of an Oil-red-O staining to assess β-VLDL, acLDL or oxidized LDL (oxLDL) uptake in either Sib-*QKI*^*+/+*^ (upper panels) or Pat-*QKI*^*+/−*^ (lower panels) macrophages that were first differentiated for 7 days with GM-CSF. Scale bar, 100 μm. Inset is a high-magnification image of lipid-droplet accumulation. Data expressed as mean±s.e.m.; Student's *t*-test; *P<0.05 and **P<0.01.

As QKI expression remarkably increased during monocyte-tomacrophage differentiation (Fig. 2c–f) and our aforementioned GO analysis revealed a strong association for changes in QKI expression and lipid metabolism (Fig. 7a), we tested whether a reduction in QKI expression influences the handling of lipids. For this, we first assessed the mRNA expression levels of a subset of established lipid-related genes in monocytes and macrophages derived from WT and *qk*^*v*^ mice. As shown in Fig. 8a, monocytes from *qk*^*v*^ mice are characterized by significant reductions in *NR1H3* (known as *LXRα*) and *PPARG (PPARγ)* expression, as well as cholesterol uptake (*CD36* and *LDLR*) and efflux (*ABCG1*) receptors, as compared with WT monocytes. These effects were diminished on conversion to macrophages (Fig. 8a).

We subsequently assessed the expression levels of these lipid metabolism/homeostasis genes in human PB-derived monocytes and macrophages (Fig. 8b and Supplementary Fig. 5). Similar to *qk*^*v*^ monocytes, Pat-*QKI*^+/*−*^ monocytes were characterized by decreased *NR1H3* and *PPARG* expression, as well as LDLR and *SCARB1* (Fig. 8b). In contrast to *qk*^*v*^ monocytes, *ABCG1* expression was potently increased. Similar to *qk*^*v*^ macrophages, this differential gene expression profile appeared to normalize in Pat-*QKI*^*+/−*^ macrophages as compared with Sib-*QKI*^*+/+*^ macrophages (Fig. 8b). Moreover, in primary human macrophages where GapmeR-mediated knockdown of QKI was realized, we observed significant increases in *MYLIP/IDOL* and *ABCG1* expression, whereas *CD36* displayed a trend towards decreased expression (Supplementary Fig. 5).

Having identified that changes in QKI expression levels had an impact on lipid-associated gene expression, we investigated whether lipid loading affected QKI expression levels. Indeed, treatment with either acetylated low-density lipoprotein (acLDL) or β-very low-density lipoprotein (β-VLDL) led to significant increases in QKI-5 mRNA levels, while QKI-6 and QKI-7 levels also increased, albeit not significantly (Fig. 8c). In contrast to primary monocytes and macrophages, THP-1 ‘monocytes' did not display significant changes in lipid metabolism gene expression. However, as shown in Fig. 8d, treatment with modified LDL increased expression of cholesterol uptake genes (*CD36* and *VLDLR*), along with significant increases in cholesterol efflux genes (*ABCA1* and *ABCG1*). Taken together, these studies suggested that changes in QKI expression could have an impact on the net balance of genes that control lipid metabolism and homeostasis.

Finally, we tested whether these QKI-mediated changes in lipid-associated gene expression could translate into consequences for lipid uptake and foam cell formation, a phenomenon tightly associated with pro-inflammatory macrophage function^7^. As shown in Fig. 8e, the impact of decreased QKI expression on foam cell formation on loading with β-VLDL was clear, as sh-QKI THP-1 ‘macrophages' displayed less extensive lipid staining as compared with sh-Cont THP-1 ‘macrophages' (Fig. 8e). Similarly, in Pat-*QKI*^*+/−*^ macrophages we observed significantly less lipid staining after β-VLDL treatment (Fig. 8f). Even more striking was the potent decrease in oxidized LDL (oxLDL) loading, an atherosclerosis-relevant antigen, in Pat-*QKI*^+/*−*^ macrophages (Fig. 8f). Collectively, these studies strongly suggested that the posttranscriptional processing of (pre-) mRNA transcripts by QKI is essential for the physiologic functioning of monocytes and macrophages in disease settings such as atherosclerosis.

## Discussion

**Figure 9:**
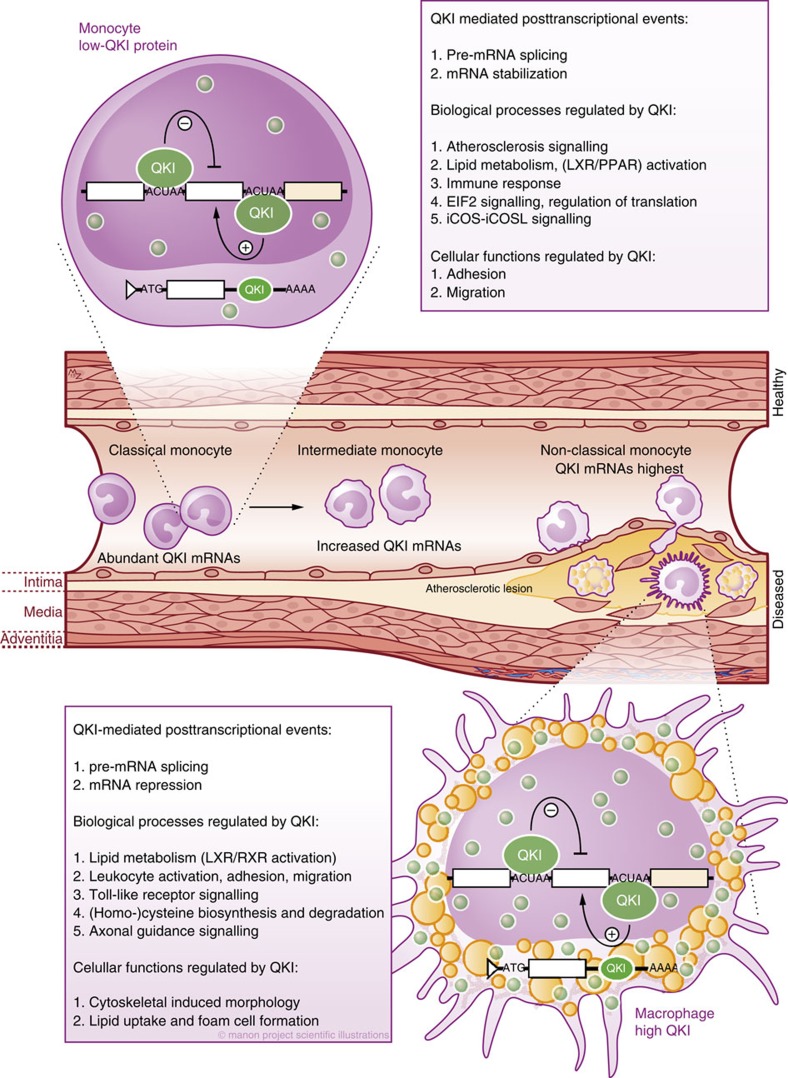
Schematic depicting how QKI posttranscriptionally regulates monocyte to macrophage differentiation and atherosclerosis development. QKI mRNA expression increases in intermediate monocytes, reaching a peak in the non-classical monocyte (middle). Monocytes adhere to the endothelium at sites of tissue injury, leading to their activation and migration into the subendothelial space. This process requires QKI, as the targeted ablation of QKI impaired monocyte adhesion and migration, and the evident transition in cellular phenotype requires extensive reprogramming of the posttranscriptional landscape. On tissue entrance, the monocyte differentiates into a macrophage, a conversion that was associated with a potent increase in QKI protein. This increase potentiates the interaction of QKI with (pre-)mRNA targets, enhancing splicing and target mRNA repression. The loss of QKI in macrophages results in an inability to adopt the macrophage phenotype and a perturbation of lipid uptake and foam cell formation.

Genes involved in regulating the transition of monocytes into pro-inflammatory macrophages serve as excellent therapeutic targets for limiting the progression of inflammation-driven diseases such as rheumatoid arthritis and atherosclerosis^3^,^6^. Our data indicate that alongside wide-ranging changes in gene expression, the differentiation of monocytes to macrophages requires extensive alternative splicing of pre-mRNA species and pinpoint QKI as a novel posttranscriptional regulator of both of these processes (Fig. 9).

Expression of the transcription factor PU.1 is associated with the activation of gene expression profiles that drive the differentiation of CD34^+^ haematopoietic progenitor cells towards a myeloid fate, including monocytes and macrophages^46^,^47^. Recent work by Pham *et al*.^48^ identified that the binding of PU.1 appears to be enhanced by cooperativity with neighbouring transcription factor binding sites, such as KLF4. Importantly, PU.1 induces the expression of critical monocyte transcription factors, including KLF4 (ref. 49). Of note, KLF4 has been demonstrated to bind to the QKI promoter region of VSMCs, which is GC rich^50^. Furthermore, chromatin immunoprecipitation sequencing data derived from HL-60 cells embedded in the UCSC Encode browser revealed two experimentally determined PU.1-binding sites in the QKI promoter^51^ (Supplementary Fig. 6). Collectively, these findings suggest that PU.1, potentially in concert with KLF4, could be responsible for driving QKI expression during monocyte-to-macrophage differentiation.

Our findings also suggest that the abundant expression of QKI mRNAs in naive monocytes could serve to prime these cells with the capacity to rapidly respond to pro-inflammatory triggers at sites of injury. Although we did not assess the translational kinetics of QKI mRNA into protein, our investigation of monocyte activation, adhesion and differentiation strongly suggests that the determination of pre-mRNA fate by QKI critically has an impact on the capacity of the monocyte to aid in response to vascular injury. To date, the genome-wide (alternative) splicing patterns during monocyte-to-macrophage differentiation had not been described. However, the consequences of many splicing events herein described, such as γ-adducin (*ADD3*), *FCGR2B* and *VLDLR* are unknown. However, given that phosphorylation of the γ-adducin C terminus triggers dissociation from spectrin and cortical actin loss^52^, it is plausible that the QKI-mediated exclusion of a 13 amino acid coding exon proximal to this region could have an impact on cytoskeletal dynamics in monocytes and macrophages. Furthermore, alternative splicing of exon 6 in *FCGR2B* could potentially have an impact on the inhibitory role of this protein in monocyte- and macrophage-mediated phagocytosis at sites of vascular injury^53^. Therapeutic strategies tailored to target such splicing events in monocytes could potentially deter their conversion to disease-accelerating macrophages.

The expanding repertoire of RNA species have led to the emergence of RNA-based therapeutics as a novel means of treating both rare and common diseases, such as muscular dystrophy and cancer, respectively^54^,^55^. This is based on extensive efforts geared towards identifying how changes in coding (alternative splicing) and non-coding RNAs (miRNA and long-non-coding RNA) have an impact on cellular pathophysiology^56^. Importantly, the fate of these coding and non-coding RNAs at the cellular level are determined primarily by the more than 500 RBPs that regulate eukaryotic cell biology^32^. Recently, the RNA motifs to which a significant portion of these RBPs bind has been characterized^32^, enabling the systematic identification of RNA targets for a given RBP within cells, including QKI^29^,^30^,^31^,^32^. Our genome-wide evaluation of posttranscriptional events mediated by QKI implicate both direct and indirect posttranscriptional roles for this protein, where the absence of ACUAA motifs or QREs could nonetheless involve QKI, potentially by tethering to other RBPs, or through QKI-mediated changes in the expression of other RBPs^57^. Moreover, in spite of the presence of QREs within target mRNAs, monocytes and macrophages probably express a large variety of RBPs that compete with QKI for access to either identical or similar motifs with varying affinities within a short stretch of RNA nucleotides^19^, which could preclude the observation of a posttranscriptional event.

In conclusion, we have identified QKI as a critical posttranscriptional regulator of pre-mRNA splicing and transcript abundance in monocytes and macrophages. We propose that the RBP-induced reprogramming of the posttranscriptional landscape could generate novel targets for the effective attenuation of inflammatory diseases.

*Note added in proof:* After the acceptance of our paper, we were informed by Dehghan *et al*.^58^ of the CHARGE Consortium's identification that single nucleotide polymorphisms proximal to QKI significantly associated with myocardial infarction and coronary heart disease risk.

## Methods

### Human immunohistochemistry studies

Early lesions are defined as fatty streaks or PIT, whereas advanced plaques consist of both FCA and IPH (fibroatheroma with early-stage or late-stage necrotic core). Scoring of plaque stage, based on characteristics such as thin cap fibroatheroma (vulnerable or ruptured plaques), vascularization, IPH and/or thrombi/fibrin deposits, were scored by a trained pathologist.

Paraffin tissue sections from human carotid arteries were deparaffinized and rehydrated. After pre-treatment with TE buffer (pH 9 for QKI-5 and QKI-7) or citrate buffer (pH 6 for QKI 6) for antigen retrieval, sections were incubated overnight at 4 °C with primary mouse-anti-human pan-QKI (10 μg ml^*−*1^, clone N147/6; UC Davis/NIH NeuroMab Facility, Davis, CA, USA), mouse anti-human QKI-5 (10 μg ml^*−*1^, clone N195A/16; NeuroMab/Antibodies, Inc.); mouse anti-human QKI-6 (10 μg ml^*−*1^, clone N182/17; NeuroMab/Antibodies Inc.) or mouse anti-human QKI-7 (10 μg ml^*−*1^, clone N183/15; NeuroMab/Antibodies Inc.) diluted in Tris-buffered saline containing 1% BSA and 0.1% Tween 20. Subsequently, sections were washed in Tris-buffered saline and incubated with a secondary biotinylated sheep-anti-mouse antibody (GE Healthcare, Eindhoven, The Netherlands). Next, the sections were incubated with streptavidin ABC-alkaline phosphatase (Vector Laboratories, Peterborough, UK) and colour was developed using the Vector Red staining kit (Vector Laboratories), followed by haematoxylin counterstaining. No primary antibody was used for the negative control. QKI/CD68 co-localization immunostaining was achieved using CD68 (Dako-KP1, DakoCytomation) and pan-QKI antibodies, with CD68 and QKI being counterstained with Vector Blue and Vector Red, respectively. For quantification, slides were analysed in a blinded manner using a Leica DM3000 light microscope (Leica Microsystems) coupled to computerized morphometry (Leica Qwin 3.5.1).

### BM transplantation

Male *LDLR*^*−*/*−*^ mice were housed in sterile filter-top cages and fed a chow diet (Special Diet Services, Witham, Essex, UK). Drinking water was infused with antibiotics (83 mg l^*−*1^ ciprofloxacin and 67 mg l^*−*1^ polymyxin B sulfate) and 6.5 g l^*−*1^ sugar and was provided *ad libitum*. BM transplantation studies were performed as previously described with minor modifications^59^. Briefly, to induce BM aplasia, 10- to 12-week-old recipient mice were exposed to a single dose of 9 Gy (0.19 Gy min^*−*1^, 200 kV, 4 mA) total body irradiation, using an Andrex Smart 225 Röntgen source (YXLON International, Copenhagen, Denmark) with a 6-mm aluminium filter, 1 day before transplantation. After 24 h, BM cell suspensions were prepared by flushing the femurs of ∼12-week-old *qk*^*v*^ mice or age-matched LM controls (C57/Bl6-J background; Jackson Laboratories, Bar Harbor, USA) with PBS, after which 5 × 10^6^ cells were injected into the tail vein of recipient mice. After 8 weeks of recovery, the mice were placed on a Western-type diet, containing 0.25% cholesterol and 15% cacao butter (Special Diet Services) for 8 weeks (*n*=12 per group). Immunohistochemical analysis and quantification of the aortic root using anti-Monocyte and Macrophage-2 (MOMA2) antibody (Sigma-Aldrich) for MOMA2-positive cell area (expressed as a percentage of total plaque area), VSMC content (smooth muscle α-actin-positive cells) and collagen (picosirius red staining) was performed in a blinded manner. Haematologic chimerism of the transplanted *LDLR*^*−*/*−*^ mice was validated using the following primers: *qk* forward primer 5′-TGTGACTTGGGGACTGTCAA-3′; *qk* reverse primer 5′-AAAGGGAAAATTTAGCAACAA-3′.

BM-derived *WT* and *qk*^*v*^ monocytes were isolated using CD115^+^ antibody coupled to magnetic beads (Miltenyi Biotech, Leiden, The Netherlands) and differentiated for 7 days to macrophages using mouse recombinant M-CSF (PeproTech, Hamburg, Germany) in RPMI 1640 medium (Gibco, Bleiswijk, The Netherlands) containing 10% FCS (Bodinco, Alkmaar, The Netherlands) and 0.01 μg ml^*−*1^ glutamine, 50 units per ml penicillin and 50 μg ml^*−*1^ streptomycin.

### Lentiviral transduction of monocytes

Human THP-1 ‘monocytes' (ATCC, Manassas, VA, USA) were transduced with lentiviral particles encoding either sh-QKI or sh-Cont (catalogue number: SHC202; MISSION library, Sigma-Aldrich). Stable transductants were selected using 3 μg ml^*−*1^ puromycin (Sigma-Aldrich) for 72 h.

### GapmeR design

A single-stranded RNA–DNA hybrid antisense oligonucleotide (GapmeR) was designed to target exon 2 of QKI (Eurogentec, Maastricht, The Netherlands), along with a scrambled GapmeR control. Both the GapmeR and scrambled control were 22 nucleotides in length and consisted of RNA (A,C,G,U) and DNA (dA, dC, dG, dT) in a 6-10-6 manner, with a phosphorothioate backbone (*) and a 6-FAM label on the 5′-end. GapmeR sequences were as follows:5′-A*C*A*U*G*U*dC*dT*dT*dT*dC*dC*dG*dT*dA*dC*U*C*U*G*C*U-3′ for the QKI-targeting GapmeR and 5′-U*G*C*C*U*C*dT*dC*dT*dC*dG*dT*dA*dC*dC *dG*U*A*U*U*U*A-3′ for the scrambled control.

### Monocyte subpopulation analysis

Human monocytes were derived from healthy donor buffy coats (Ethical Approval Number BTL 10.090) following Ficoll density gradient centrifugation and isolated from the PB mononuclear cell fraction using a negative selection cocktail to isolate unlabelled monocytes (StemCell Technologies, Grenoble, France). Purified monocytes were subsequently incubated with 1 μg ml^*−*1^ CD14-FITC and 1 μg ml^*−*1^ CD16-Pc5 (Beckman Coulter, Woerden, The Netherlands) for 30 min at 4 °C and FACS sorted using a FACSCalibur (BD Biosciences, Breda, The Netherlands). RNA was isolated from the subpopulations using Trizol reagent (Thermo Fisher Scientific, Bleiswijk, The Netherlands).

### Monocyte and macrophage culture

Human monocytes were isolated from buffy coats of healthy donors with an antibody to CD14, conjugated to magnetic beads to allow for MACS sorting (Miltenyi Biotech). Cells were cultured in RPMI media supplemented with 10% FCS and 0.01 μg ml^*−*1^ glutamine at 37 °C and 5% CO_2_. Differentiation of primary CD14^+^ monocytes to pro-inflammatory macrophages was achieved by stimulating with 5 ng ml^*−*1^ GM-CSF (Thermo Fisher Scientific) or 5 ng ml^*−*1^ M-CSF (Miltenyi Biotech). Human THP-1 ‘monocytes' were cultured in RPMI media supplemented with 10% FCS and 0.01 μg ml^*−*1^ glutamine at 37 °C and 5% CO_2_, with differentiation into macrophages being induced by treating with 100 nM PMA^60^. TS/216 for integrin activation experiments was kindly provided by Dr Arnoud Sonneberg, Netherlands Cancer Institute, Amsterdam, The Netherlands. Foam-cell formation was assessed by treating sh-Cont and sh-QKI THP-1 ‘macrophages' or Sib-QKI^+/+^ and Pat-QKI^+/*−*^ macrophages for 24 h with either 25 μg ml^*−*1^ β-VLDL or 50 μg ml^*−*1^ acLDL, or 10 μg ml^*−*1^ oxLDL, after which the cells were fixed with 10% Formafix, and Oil-Red-O stained and haematoxylin counterstained. Oil-Red-O area per field of view was divided by the number of cells using ImageJ software.

### *In vitro* perfusion assay

Glass coverslips were coated with type I collagen, after which the system was perfused with platelet-rich plasma for 10 min. Next, the system was flushed with flow buffer (20 mmol l^*−*1^ HEPES, 132 mmol l^*−*1^ NaCl, 6 mmol l^*−*^ KCl, 1 mmol l^*−*1^ MgSO_4_, 1.2 mmol l^*−*1^ KH_2_PO_4_, 5 mmol l^*−*1^ glucose, 1.0 mmol l^*−*1^ CaCl2 and 0.5% BSA) for 2 min. Sh-Cont and sh-QKI THP-1 were resuspended in flow buffer at a concentration of 4 × 10^6^ ml^*−*1^, after which the cells were perfused over the substrate for 5 min at 1 dyne cm^*−*2^. Cellular adhesion was tracked visually using a Leica DMI5000 microscope. The flow rate was subsequently increased to 2 dynes cm^2^ for 2 min, followed by the visual assessment of firm adhesion of perfused monocytes for a duration of 3 min, after which photomicrographs of ten random fields of view in the fluidic chamber were taken and quantified.

### Cellular migration assays

Transwell cellular migration studies of sh-Cont and sh-QkI THP-1 ‘monocytes' towards 1 nM *N*-formyl-methionyl-leucyl-phenylalanine (fMLP, Sigma-Aldrich) or 10 ng ml^*−*1^ macrophage chemoattractant protein 1 (R&D Systems, Abingdon, UK) for human primary monocytes were performed as previously described^61^. Briefly, cell migration was assessed using Corning Transwell polycarbonate membrane cell culture inserts (6.5 mm transwell with 5.0 μm pore size, Sigma). The lower chamber was loaded with RPMI containing 0.25% BSA and desired chemoattractant. Wells containing no chemoattractant were used as negative controls. Cells were loaded in the upper chamber of the transwell inserts (10^5^ cells) and incubated overnight at 37 °C. The following day, cell migration (adherent cells) was quantified by manual counting or ImageJ.

### Gene expression microarrays

RNA was isolated from unstimulated sh-Cont and sh-QkI THP-1 ‘monocytes' (day 0), and from sh-Cont and sh-QkI THP-1 cells stimulated with 100 nM PMA for 3 days using Trizol (Thermo Fisher Scientific) and the RNeasy Mini Kit (Qiagen, Heidelberg, Germany) according to the manufacturer's instructions. RNA quantity and quality was measured using a NanoDrop spectrophotometer (Nanodrop Technologies, Wesington, USA) and an Agilent 2100 bioanalyser (Agilent Technologies, Santa Clara, USA). Samples meeting a RNA integrity number critieria of >8 were used for further analysis.

### Splicing microarrays

Microarray data is deposited in GEO under the accession number GSE74887. Targets were prepared from three replicate cultures for each sample. Complementary DNA synthesis and amplification was performed using the WT Expression Kit (Ambion, Bleiswijk, The Netherlands). Samples were enzymatically fragmented and biotinylated using the WT Terminal Labeling Kit (Affymetrix, Santa Clara, California, USA). Labelled target was hybridized to the HJAY Chip (Affymetrix 540091). Chips were washed and scanned using the Fluidics Station 450 and Affymetrix Gene ChIP Scanner 3000 7G (Affymetrix). Data were analysed as previously described^62^. Briefly, in the absence of mismatch probes on these microarrays, probe intensities were first used to construct an empirical CDF, which was subsequently used to calculate an empirical *P*-value that a particular probe's intensity arose from the background of all probes. Probes were stratified for GC content (thermodynamically favourable GC base pairing). For each probe set, the median *P*-value of the set of individual probes in the probe set was used as the *P*-value for that probe set. Before assessing for alternatively spliced transcripts from a particular locus, we first determined whether the gene was expressed. Next, if the expression criteria was met, we determined whether RNA containing two or more alternative splice junctions was detectable using the isoform-specific probes. In situations where the probe sets for two or more alternative isoforms were ‘present' in any sample of the data, an alternative splicing event was scored. For these events, the junction probe sets that hybridized to individual isoforms were identified and the probes they contained were used for the Kruskal–Wallis test. Next, we normalized individual probe intensities and grouped the replicate values. Subsequently, using R software, the kruskal.test function was used to test the hypothesis that the probe intensities come from identical populations. If the resulting *P*-value was small enough, the null hypothesis was rejected and the alternative hypothesis that the probes were differentially expressed was accepted. To determine an appropriate value for the 0.01 significance level, 12,740 Kruskal–Wallis tests on randomly selected probe sets were performed, yielding an *α*-value that associated with the 1% quantile of randomly selected probes (1.975486 × 10^−3^). To account for multiple testing, a Bonferroni-corrected *α*-value of 1.975486 × 10^−3^/1.2740 × 10^4^=1.550617 × 10^−7^ was used as a *P*-value cutoff for significance. The Sepscore is log2 (Include/Skip ratio) of the experimental sample over the reference sample. Exon inclusion generates a positive Sepscore, whereas exon exclusion generates a negative Sepscore.

### Word (5-mer) enrichment and positional mapping

Counts of all 5-mers (ACUAA) in the selected region of an exon set are compared with their counts from a background set of sequences using Fisher's exact test, with multiple testing correction. For motif mapping, we plotted the frequency of specific motifs in 50-nucleotide windows slid along the intron sequences upstream and downstream of each selected exon set with 5-nucleotide sampling intervals. At each interval, we computed the average number of motifs in exons activated or repressed in shRNA-treated cells, and background exons in the same experiment with no splicing change. For the background set, we empirically estimated the 95% confidence interval of motif frequency (error bars). For peaks of consecutive points outside the 95% interval, we applied the Mann–Whitney–Wilcoxon test to estimate a *P*-value that the motif frequency at each point within the peak is greater than background. As the points were not independent, we estimated a *q*-value for each peak by finding the most significant *P*-value for any point in the peak and applying Bonferroni correction for the number of points within the peak. A second Bonferroni correction controlled for the number of possible positions at which a peak might occur, yielding the final *q*-values. To explain further; the effect of QKI depletion on every assessed exon was calculated from the RNA-seq or the splicing-sensitive microarray: that is, whether a reduction in QKI causes inclusion or exclusion from the final mature mRNA transcript.

Next, the intronic regions around differentially spliced exons were analysed for the presence of ACUAA motifs (5-mers). By doing this for every alternatively spliced exon, we could detect an enrichment of ACUAA motifs in the upstream introns of the exons that were included in a ‘QKI-deficient' cell: QKI is not binding upstream; thus, the exon is included, as measured by RNA-seq or splicing-sensitive microarray.

In contrast, we could detect an enrichment of ACUAA motifs in the downstream introns of the exons that are included on QKI reduction. QKI binding in the downstream intron would normally give inclusion, but now QKI cannot bind downstream; thus, exclusion is now favoured as assessed by the RNA-seq or splicing-sensitive microarray.

### RNA-seq library preparation

RNA-seq data are deposited in GEO under the accession number GSE74979. For each sample, the non-ribosomal fraction of 3 μg of total RNA was isolated using a Ribo-Zero rRNA removal Kit (Epicentre, Madison, Wisconsin, USA). Ribo-Zero-treated RNAs were used to generate barcoded cDNA libraries using the TruSeq RNA Sample Preparation kit (Illumina), with the following additions. Size selections were performed before and after cDNA amplification on an E-gel Safe Imager (Invitrogen) using 2% E-gel SizeSelect gels (Invitrogen). The cDNA fraction of 300 bp in size (including adapters) was isolated and purified. Indexed libraries were pooled and sequenced (paired-end 50 or 100 bp reads) on the Illumina HiSEQ platform to a depth of 41–70 million reads per sample (QB3 Vincent J. Coates Genomics Sequencing Laboratory). After removal of PCR duplicates and repeats, there were 21–26 million uniquely mapping paired-end reads (37–61%).

### Mapping and analysis of RNA-seq data

All samples were mapped using Tophat2 (refs 63, 64) with Bowtie2 (ref. 64) as the underlying alignment tool. The input Illumina fastq files consisted of paired-end reads with each end containing 100 bp (except for 2 samples with 50 bp of paired-end reads). For equivalency, 100-bp reads were trimmed to 50 bp before mapping. The target genome assembly for the human samples was GRCh37/UCSC-hg19 and Tophat was additionally supplied with a gene model (using its ‘-GTF' parameter) with data from the hg19 UCSC KnownGenes track^65^. For multiple-mapped fragments, only the highest scoring mapping determined by Bowtie2 was kept. Only mappings with both read ends aligned were kept. Potential PCR duplicates (mappings of more than one fragment with identical positions for both read ends) were removed with the samtools ‘rmdup'^66^ function, keeping only one of any potential duplicates. The final set of mapped paired-end reads for a sample were converted to position-by-position coverage of the relevant genome assembly using the bedtools ‘genomeCoverageBed' function^67^. To determine the count of fragments mapping to a gene, the position-by-position coverage was summed over the exonic positions of the gene. This gene total coverage was divided by a factor of 100, to account for the 100-bp of coverage induced by each mapped paired-end fragment (50 bp from each end) and rounded to an integer. This was calculated for each gene in the UCSC Known Gene set. For input to DESeq^68^, all genes with non-zero counts in any sample were considered. Two replicates of each sample were combined per the DESeq methodology. SpliceTrap^69^ was used to analyse splicing changes with the parameters j15, ch0.1, ir0.1 and IRMyes.

For mapping of reads that are chimeric to the reference genome^70^, to identify the translocation breakpoint on chr6 we used STAR-Fusion https://github.com/STAR-Fusion/. Non-splice junction reads from the macrophage samples that mapped from the QKI gene on chr6 to any location on chr5 were inspected and several were found, which mapped from QKI intron 4 to a site that is strongly transcribed from chr5 in the patient but not at all in her sibling.

### Genome-wide computational analysis for RNA motifs

Human mature mRNA sequences were downloaded from UCSC RefSeq database (hg19). Computational screening was performed for the QRE motif, UACUAAY N1-20 UAAY, and counts calculated for the longest annotated transcript. After cross-referencing these transcripts with either the transcripts annotated on the microarray or the RNA-seq, we annotated the number or QREs in the Supplementary Data. Transcripts of which we were unable to assess whether they contain one or more QREs, we annotated as NA and these were excluded from analysis to generate the CDF plots in Figs 3j and 5h. To generate the Venn diagrams and scatterplots for the RNA-seq of monocytes and macrophages (Fig. 3g,i), a ±1.5-fold change cutoff was applied together with a minimal expression cutoff of patient+sibling ≥1 CPM, to avoid artificially large fold changes due to very low expression values. To generate the Venn diagrams and scatterplots for the THP-1-derived expression data (Fig. 5e, g), we applied a ±1.5-fold change cutoff and applied a DESeq-derived *q*-value cutoff of *q*≤0.05.

### MISO analysis

Mixture of isoforms (MISO) analysis was used to assess, quantify and visualize alternative transcripts based on RNA-seq data. Sequences obtained from RNA-seq were aligned using TopHat2 to the human genome v19 transcriptome (annotation-set kindly provided by Dr Christopher Burge, MIT, Cambridge, USA). Using the alignment files (BAM files), MISO analyses was performed on our RNA-seq paired-end sequencing data to identify alternative splicing events, as previously described^71^. Second, an annotation set containing only exons surrounding the splicing event were included in the analysis to generate a more in-depth analysis of select splicing events. A pairwise comparison was performed using the sibling and patient monocyte and macrophage on both the full and selected annotation sets. Additional visualization was performed using the sashimi plot subpackage from MISO, while modifications in the plotting procedure were made to allow visualization of supplementary annotation tracks including ACTAA and QKI PAR-CLIP sites, along with RefSeq transcripts that define the event at (https://github.com/wyleung/rnaveer).

### Western blot analysis

Polyacrylamide gel electrophoresis was used to resolve proteins from cellular lysates harvested in RIPA buffer. Protein determinations (BCA) were performed to ensure equal loading of protein on a per-sample basis. QKI-5, -6 and -7 were detected using primary mouse monoclonal antibodies that target pan-QKI (1:1,000, N73/168; UC Davis/NIH NeuroMab Facility), QKI-5 (1:1,000, N195A/16; UC Davis/NIH NeuroMab Facility), QKI-6 (1:1,000, N182/17; UC Davis/NIH NeuroMab Facility) or QKI-7 (1:1,000, N183/15.1; UC Davis/NIH NeuroMab Facility), or polyclonal antibodies targeting QKI-5 (1:1,000, AB9904; Millipore, Amsterdam, The Netherlands), QKI-6 (1:2,000, AB9906; Millipore) and QKI-7 (1:2,000, AB9908; Millipore). For loading references, rabbit polyclonal antibodies were used to detect β-actin or Histone H3 (both 1:4,000; Abcam, Cambridge, UK). All gels were run and blotted with Bio-Rad TGX pre-cast gels and blotted on nitrocellulose 0.2 μm using the Bio-Rad TurboBlot system (Bio-Rad Laboratories). Full blots are shown in Supplementary Fig. 7.

### Quantification of pre-mRNA expression levels by PCR

RNA was harvested from monocytes and macrophages using Trizol reagent (Thermo Fisher Scientific). Standard mouse and human cDNA was made using oligo-dT primers (Invitrogen), whereas cDNA for alternative splicing studies was synthesized using random primers (Invitrogen). Primer sets designed for specific pre-mRNA amplification by quantitative RT–PCR analysis are provided in Supplementary Table 1. Quantitative RT–PCR analysis for designated mRNA products was performed using SYBR Green master mix (Bio-Rad, Veenendaal, The Netherlands). For optimal resolution of pre-mRNA splicing patterns, samples were run on an Agilent 2100 bioanalyser (Agilent), with full images provided in Supplementary Figs 8–10.

### Statistics

For all experiments, *N* defined the number of biological replicates. All *in vitro* and *in vivo* results were analysed using GraphPad software with either a Student's *t*-test or analysis of variance (with a Bonferonni post test being used). All results are expressed as mean±s.e.m. Differences in *P*-values<0.05 or <0.01 were considered significant and indicated as follows: *P<0.05 or **P<0.01, respectively.

### Ethics

Informed consent was obtained for all patient-derived samples. Approval for these studies was provided by the relevant medical ethics committees, namely Maastricht University Medical Center, The Netherlands (Professor Dr E.A.L. Biessen), for immunohistochemical studies, and Leuven University Hospital, Belgium (Professor Dr H. Van Esch), for the QKI haploinsufficient and control materials. All mouse experiments were approved by the regulatory authorities of the Leiden University and were in compliance with the Dutch Government Guidelines.

## Additional information

**Accession code:** RNA sequencing and microarray data were deposited in NCBI's Gene Expression Omnibus (GEO) under accession codes GSE74979 (for RNA-seq data) and GSE74887 (for the splicing-sensitive microarray data).

**How to cite this article:** de Bruin, R. G. *et al*. Quaking promotes monocyte differentiation into pro-atherogenic macrophages by controlling pre-mRNA splicing and gene expression. *Nat. Commun.* 7:10846 doi: 10.1038/ncomms10846 (2016).

## Supplementary Material

Supplementary Information Supplementary Figures 1-10, Supplementary Table 1 and Supplementary Reference

Supplementary Movie 1 Initial adhesion of sh-Cont THP-1 ‘monocytes' in an *in vitro* cell perfusion system. Glass coverslips were coated with type I collagen, after which the system was perfused with platelet-rich plasma for 10 minutes, leading to the deposition of effector molecules to which the monocyte can adhere. This movie displays the perfusion of sh-Cont THP-1 ‘monocytes' over this bio-active substrate, leading to their attachment to the surface. Total cellular perfusion time was 5 minutes with a flow rate of 1 dyne cm-2. The movie is representative of at least three perfusions.

Supplementary Movie 2 Initial adhesion of sh-QKI THP-1 ‘monocytes' in an *in vitro* cell perfusion system. Glass coverslips were coated with type I collagen, after which the system was perfused with platelet-rich plasma for 10 minutes, leading to the deposition of effector molecules to which the monocyte can adhere. This movie displays the perfusion of sh-QKI THP-1 ‘monocytes' over this bio-active substrate, leading to a reduction in their attachment to the surface as compared to that seen for sh-Cont THP-1 ‘monocytes' in Supplementary Movie 1. Total cellular perfusion time was 5 minutes with a flow rate of 1 dyne/cm2. The movie is representative of at least three perfusions.

Supplementary Data 1 Hematologic profile of whole blood harvested from LDLR-/- mice 16 weeks after transplantation with bone marrow from C57Bl6 control (WT littermates) and quaking viable (qkv) mice (8 week recovery and 8 weeks high-fat diet).

Supplementary Data 2 RNA-seq derived mRNA abundance as CPM after quantile normalization in Sib-QKI+/+ and Pat-QKI+/- PB monocytes and macrophages.

Supplementary Data 3 RNA-seq profiling of alternative splicing events in Sib-QKI+/+ and Pat-QKI+/- PB monocytes and macrophages.

Supplementary Data 4 ACUAA motif enrichment analysis based on the splicing-sensitive microarray and RNA-seq data.

Supplementary Data 5 Microarray profiling of mRNA abundance in sh-Cont and sh-QKI THP-1 ‘monocytes' and ‘macrophages'.

Supplementary Data 6 Splicing-sensitive microarray analysis of sh-Cont and sh-QKI THP-1 ‘monocytes' and ‘macrophages' and RNA motif analysis for alternative splicing events observed in sh-Cont and sh-QKI THP-1 ‘monocytes' and ‘macrophages'.

Supplementary Data 7 Ingenuity(r) Pathway Analysis (IPA) of THP-1 and PB monocytes and macrophage datasets.

## Figures and Tables

**Table 1:** IPA assessment of pre-defined canonical pathways affected by changes in QKI expression.

Monocytes			Macrophages		
THP-1 sh-QKI versus sh-Cont			THP-1 sh-QKI versus sh-Cont		
Affected canonical pathway	−Log (*P*-value)	Affected genes	Affected canonical pathway	−Log (*P*-value)	Affected genes
Atherosclerosis signalling	9.2	*CXCL8*, *APOE*, *ICAM1*, *PDGFA*, *PLA2*, *G4C*, *CCR2*, *F3*, *LYZ*, *CCL2*, *ORM1*, *APOC1*, *IL1B*, *ORM2*, *PDGFD*, *TNF*	Superpathway of cholesterol biosynthesis	10.6	*FDPS*, *PDFT1*, *EBP*, *DHCR7*, *ACAT2*, *IDI1*, *HSD17B7*, *MSMO1*, *HMGCS1*, *CYP51A1*
Superpathway of cholesterol biosynthesis	8.2	*MVD*, *FDPS*, *CHCR7*, *ACAT2*, *HSD17B7*, *MSMO1*, *HMGCS1*,*CYP51A1*	Cholesterol biosynthesis I, II, and III	8.1	*FDFT1*, *EBP*, *DHCR7*, *DHCR24*, *HSD17B7*, *MSMO1*, *CYP51A1*
LXR/RXR activation	7.4	*SCD*, *APOE*, *LYZ*, *ORM1*, *CCL2*, *APOC1*, *IL1B*, *ORM2*, *CD14*, *PTGS2*, *IL1RAP*, *TNF*, *CYP51A1*	Superpathway of gernanylgeranylphosphate Biosynthesis I	4.4	*FDPS*, *ACAT2*, *IDI1*, *FNTB*, *HMGCS1*
Hepatic fibrosis/hepatic stellate cell activation	6.1	*CXCL8*, *APOE*, *ICAM1*, *PDGFA*, *PLA2*, *G4C*, *CCR2*, *F3*, *LYZ*, *CCL2*, *ORM1*, *APOC1*, *IL1B*, *ORM2*, *PDGFD*, *TNF*	LXR/RXR activation	4.4	*SCD*, *FDFT1*, *LYZ*, *IL1A*, *LDLR*, *IL36RN*, *NR1H3*, *IL6*, *CLU*, *CYP51A1*, *IL36B*, *AGT*
PPAR signalling	5.8	*PPARG*, *JUN*, *PPARD*, *PDGFA*, *MRAS*, *IL1B*, *PTGS2*,*PDGFD*, *TNF*, *IL1RAP*	Altered T-cell and B-cell signalling in rheumatoid arthritis	4.3	*IL1A*, *CSF1*, *IL36RN*, *TLR6*, *TLR8*, *TLR7*, *IL6*, *CSF2*, *IL36B*, *IL17A*
**RNA-seq Pat-QKI versus Sib-QKI**			**RNA-seq Pat-QKI versus Sib-QKI**		
**Affected canonical pathway**	**−Log (*****P*****-value)**	**Affected genes**	**Affected canonical pathway**	**−Log (*****P*****-value)**	**Affected genes**
T-cell receptor signalling	8.9	*CD247*, *PTPN7*, *CAMK4*, *PRKCQ*, *CD3E*, *PLCG1*, *CD8A*, *CD3D*,*CD8B*, *CD28*, *CD3G*, *LCK*, *TXK*, *ZAP70*, *ITK*	Granulocyte adhesion and diapedesis	4.9	*CXCL8*, *IL1A*, *HRH2*, *MMP7*, *SDC1*, *PPBP*, *ITGA6*, *RDX*, *CCL24*, *CCL17*, *MMP2*, *CCL22*, *C5*, *FPR1*, *CCL13*, *ICAM2*, *IL1RN*, *MMP19*, *ITGA4*
CCR5 signalling in macrophages	7.8	*CD247*, *CD3G*, *CCR5*, *CAMK4*, *PRKCQ*, *CCL4*, *CD3E*, *PLCG2*, *PLCG1*, *CCL3*, *CD3D*, *GNG10*	Agranulocyte adhesion and diapedesis	4	*CXCL8*, *MMP7*, *IL1A*, *PPBP*, *ITGA6*, *RDX*, *CCL24*, *CCL17*, *MMP2*, *CCL22*, *C5*, *MYL9*, *CCL13*, *ICAM2*, *IL1RN*, *PODXL*, *MMP19*, *ITGA4*
Role of NFAT in regulation of the immune response	7	*CD247*, *CAMK4*, *PRKCQ*, *CD3E*, *GCER1A*, *PLCG1*, *CD3D*, *GNG10*, *CD28*, *CD3G*, *LCK*, *GNAT1*, *PLCG2*, *ZAP70*, *FCGR3A*/*GCGR3B*, *FCGR1B*, *ITK*	Toll-like receptor signalling	3	*MAP2K6*, *IL1A*, *TICAM2*, *IL1RN*, *TLR7*, *MAPK13*, *TLR3*, *IRAK2*, *TRAF1*
EIF2 signalling	5.8	*RPL24*, *RPL36A*, RPS3A, *RPS27*, *RPL17*, *RPS18*, *RPS10*, *RPL39*, *RPL12*, *RPL7A*, *RPL7*, *RPL9*, *RPS28*, *RPL23A*, *RPL39L*, *RPSA*	Cysteine biosynthesis/homocysteine degradation	2.9	*CBS/CBSL*, *CTH*
iCOS-iCOSL signalling in T-helper cells	5.7	*CD247*, *CD3G*, *CD28*, *LCK*, *CAMK4*, *PRKCQ*, *CD3E*, *ZAP70*, *PLCG1*, *CD3D*, *ICOSLG*/*LOC102723996*, *ITK*	Axonal guidance signalling	2.9	*SLIT3*, *ERAP2*, *MMP7*, *SLIT1*, *PDGFA*, *SEMA6A*, *BCAR1*, *TUBB2B*, *EPHB1*, *TUBA8*, *MYSM1*, *PRKAR1B*, *GNB1L*, *WNT5B*, *ITGA4*, *SEMA3G*, *PAK4*, *ADAM15*, *TUBA4A*, *MMP2*, *KEL*, *MYL9*, *FZD4*, *ADAM12*, *SEMA4G*, *SEMA7A*, *FZD7*

IPA, Ingenuity Pathway Analysis; QKI, Quaking.

The top five affected canonical pathways are shown, along with their respective –log(*P*-value) and the genes that are affected within the particular pathway. Full IPA output is provided in Supplementary Data 7.
