# Supplementary material for: Quaking promotes monocyte differentiation into pro-atherogenic macrophages by controlling pre-mRNA splicing and gene expression
Source: Nat Commun. 2016 Mar 31;7:10846. doi: 10.1038/ncomms10846 (PMC4821877; doi:10.1038/ncomms10846)
Supplement: Supplementary Information — Supplementary Figures 1-10, Supplementary Table 1 and Supplementary Reference [file ncomms10846-s1.pdf]

Supplementary Information

Supplementary Figure 1

Chimeric reads in chromosome 5

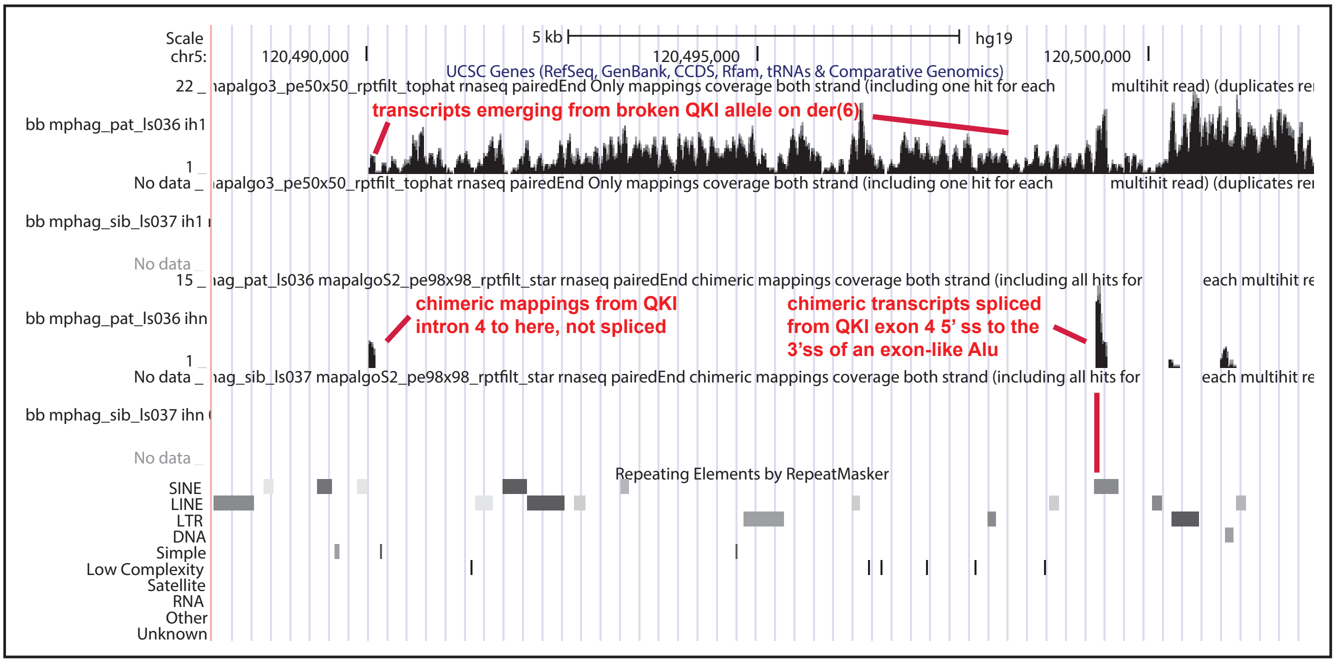

Supplementary Figure 1: RNA-seq shows chr6-derived read coverage on chr5. Genomic location according to UCSC genome browser on chromosome 5. Read coverage is only seen in patient tracks.

Supplementary Figure 2

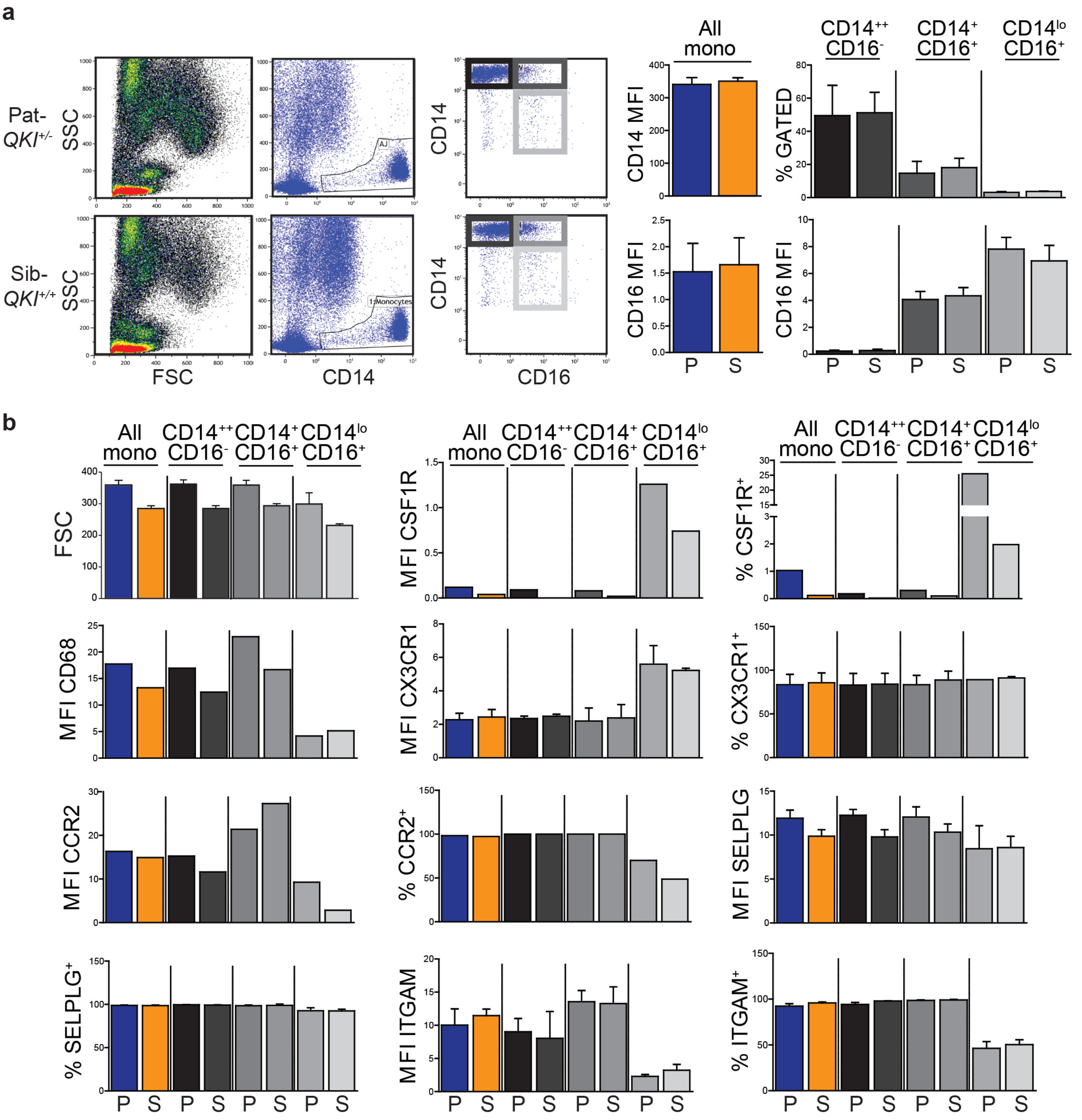

Supplementary Figure 2: FACS analysis of surface marker expression levels in monocyte subsets in the QKI haploinsufficient patient and her sibling. a. FACS analysis of PB harvested from the QKI haploinsufficient patient (P denotes Pat-QKI$^{+/-}$) as compared with an age- and sex-matched sibling control (S denotes Sib-QKI$^{+/+}$). Live cells were first gated in the FSC/SSC gate, after which monocytes were selected based in CD14/SSC expression. Monocyte subpopulations were defined as CD14^{++}/CD16^{-}, CD14^{++}/CD16^{+}, and CD14^{+}/CD16^{+}. MFI denotes mean fluorescent index. (n=1 biological replicate; n=7 technical replicates). **b.** Quantitation of average monocyte size in Pat-QKI$^{+/-}$ and Sib-QKI$^{+/+}$ monocytes based on FSC parameter. All other panels represent quantification of either MFI or percentage of gated monocytes (defined as % gene name^{+}) that express the designated surface markers at levels higher than background signal, such as CSF1R. Data are based on biological n=1, while error bars indicate technical replicates for surface markers included in more than one sample in the phenotype characterization panel (minimal n=2).

Supplementary Figure 3

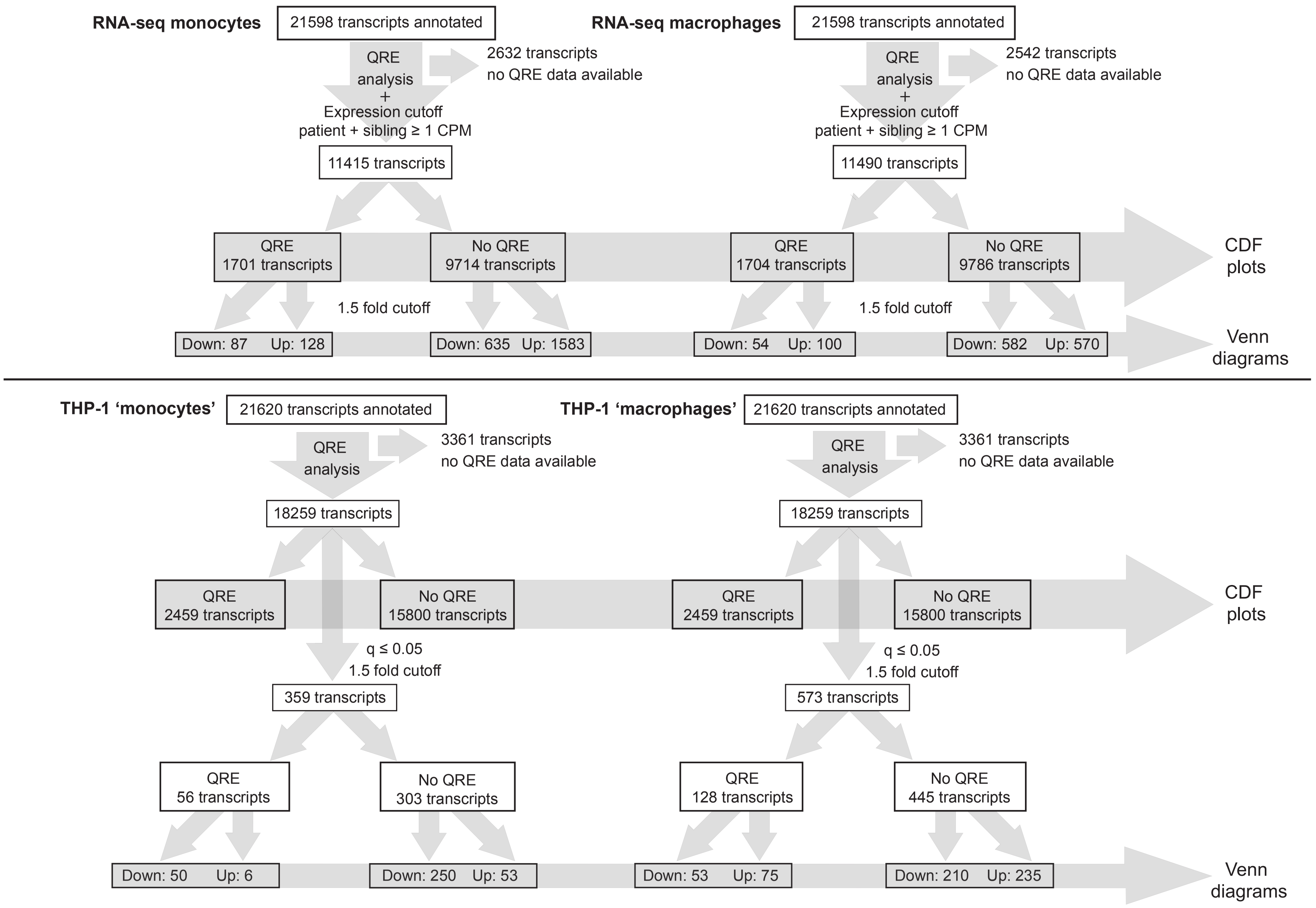

Supplementary Figure 3: Schematic representation of transcript stratification. Flow-diagram illustrating the bioinformatic approach utilized for analysis of RNA-seq data (top) and microarray-based data (bottom). Vertical arrows depict stratification based on the presence or absence of QRE's in transcripts and those achieving expression and/or significance cut-offs. Horizontal arrows indicate transcripts used to generate Venn diagrams (Fig. 3g,5e), tables of most up and downregulated genes (Fig. 3h, 5f), scatterplots (Fig. 3i, 5g) and Cumulative Distribution Fraction plots (Fig. 3j, 5h).

Supplementary Figure 4

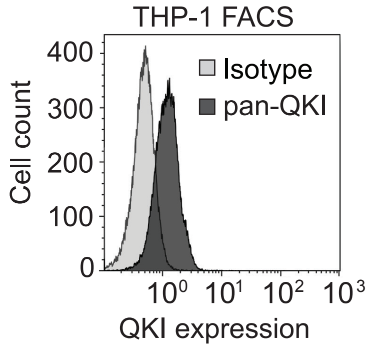

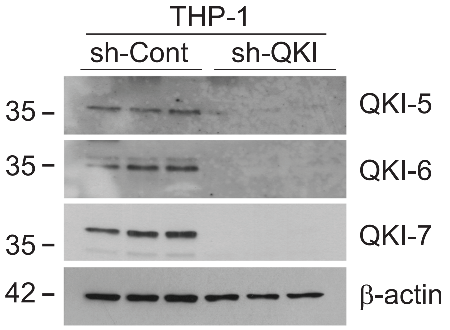

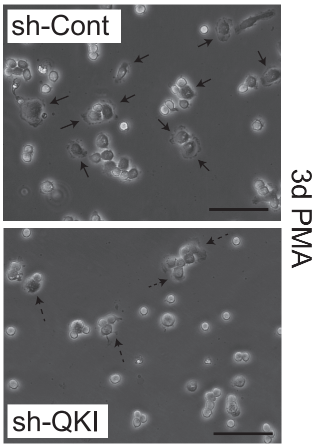

Supplementary Figure 4: QKI protein is differentially expressed during THP-1 monocyte-like to macrophage-like differentiation. a. Intracellular FACS analysis of THP-1 'monocytes' for total QKI expression. **b.** Western blot analysis of QKI-5, -6 and -7 expression in cellular lysates harvested from sh-Cont and sh-QKI transduced THP-1 'macrophages' following stimulation with PMA for 3d. **c.** Phase-contrast photomicrographs of 3 days stimulated THP-1 'macrophages'. Scale bar = 50 μm.

Supplementary Figure 5

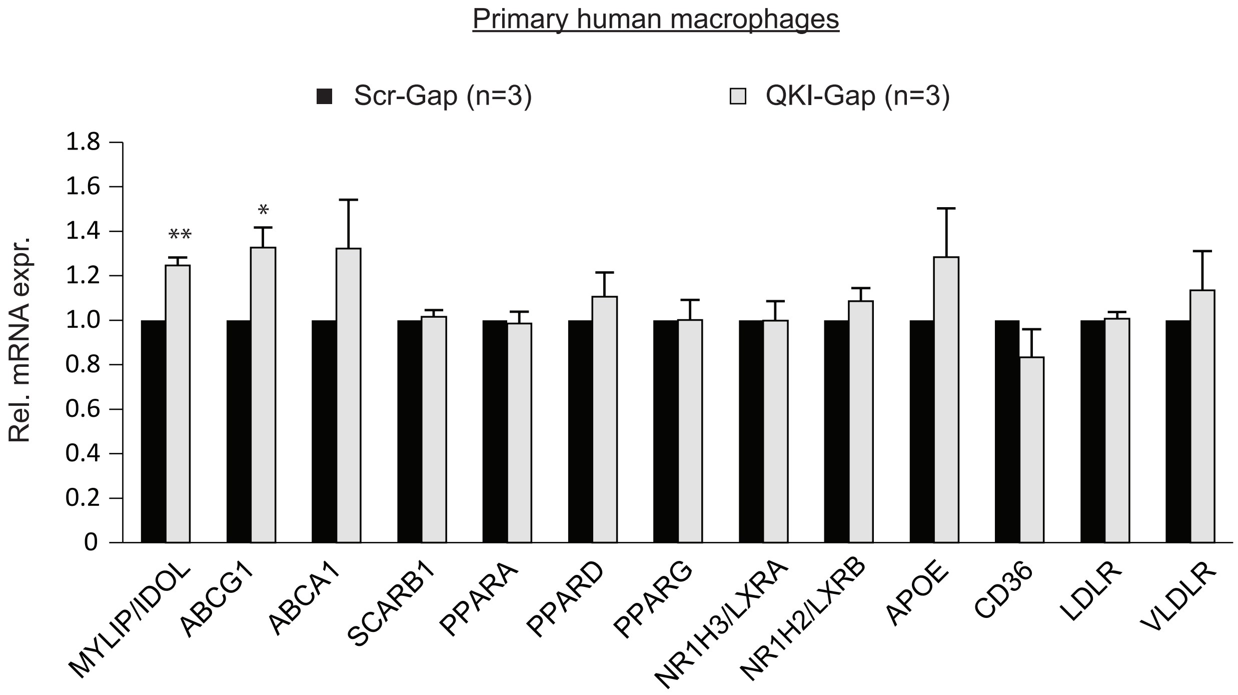

Supplementary Figure 5: GapmeR-mediated reduction of QKI expression regulates the expression of atherosclerosis-related mRNAs. qRT-PCR analysis of established atherosclerosis-related genes in QKI-Gap as compared to Scr-Gap treated macrophages. Gene expression in QKI-Gap macrophages are relative to Scr-Gap macrophages (n=3). Data expressed as mean +/- s.e.m; Student's t-test; *p<0.05, ** p<0.01.

Supplementary Figure 6

Transcription factors that bind to the QKI promoter region

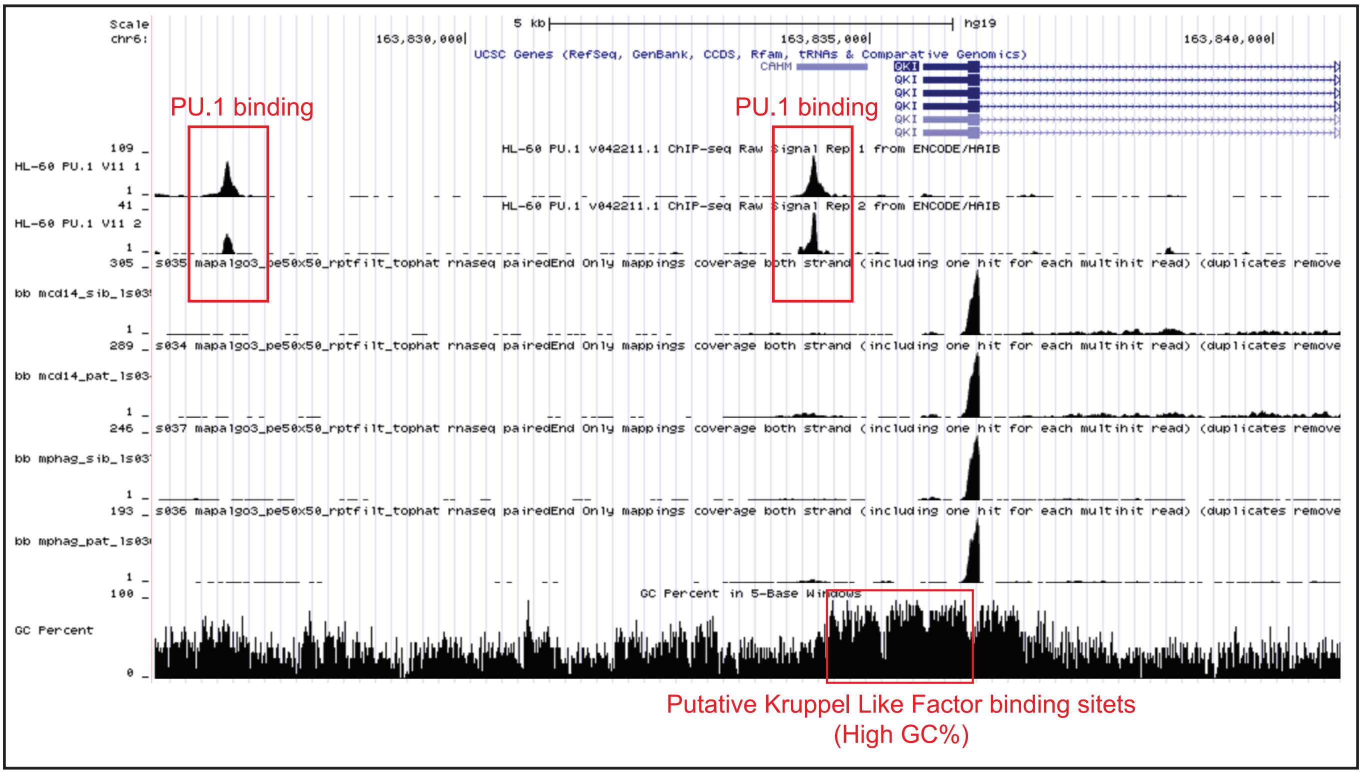

Supplementary Figure 6: Experimentally determined and putative transcription factor binding sites in the QKI promoter region. Top tracks: Genomic location and organization according to UCSC genome browser on chromosome 6 mapping to the QKI locus. Tracks 2 and 3 represent the read coverage for two independent chromatin-immunoprecipitations in HL60 cells for PU.1 [1], a well-known myeloid transcription factor. Tracks 4,5,6,7 represent read coverage for sibling and patient monocyte and macrophages. Track 8 represents percentage GC in 5 bp windows, indicative of putative Kruppel-Like Factor binding sites.

Supplementary Figure 7

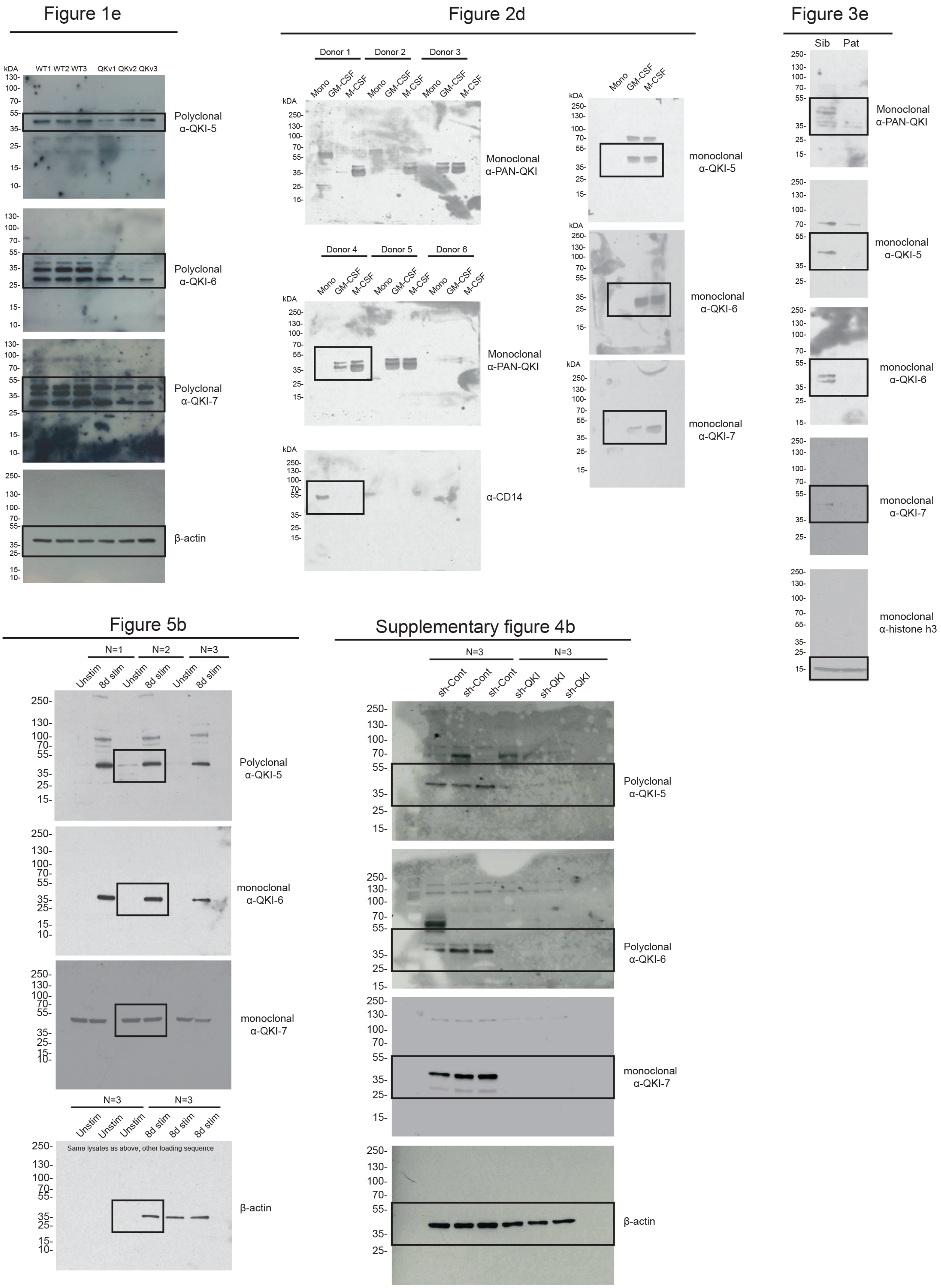

Supplementary Figure 7: Original immunoblot images utilized for preparation of figures 1e, 2d, 3e, 5b and Supplementary Fig. 4b.

Supplementary Figure 8

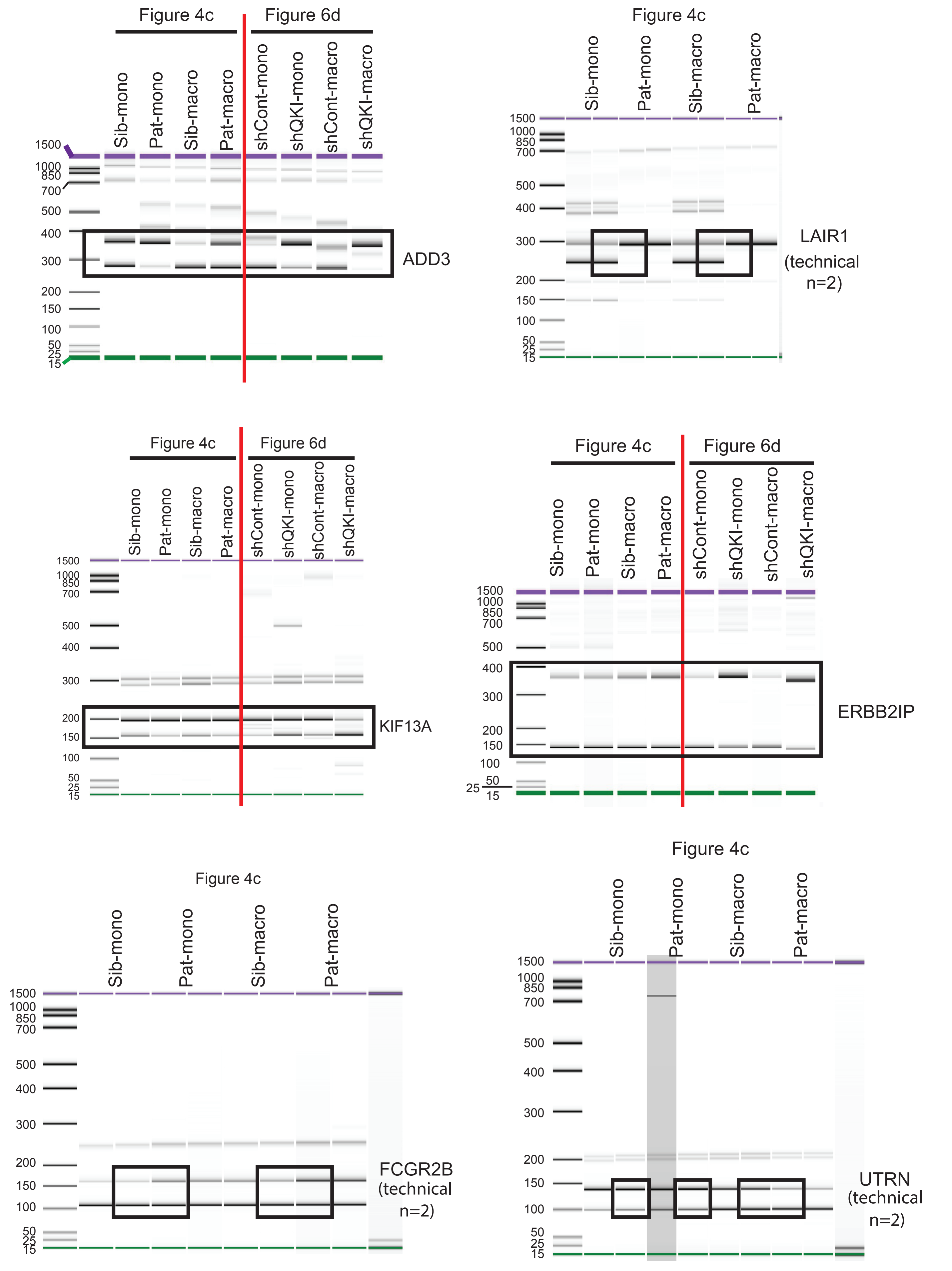

Supplementary Figure 8: Original bioanalyzer images utilized for preparation of figures 4c and 6d.

Supplementary Figure 9

Figure 6d

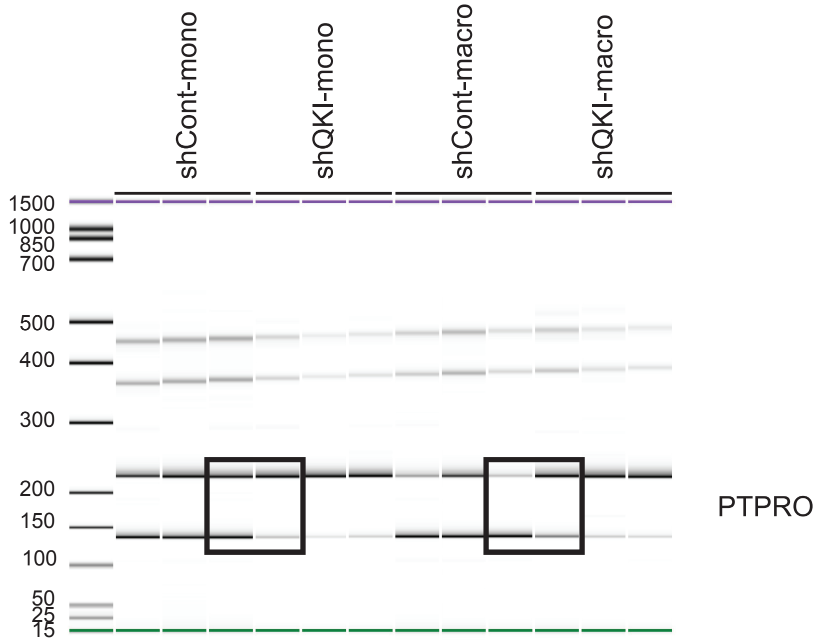

Figure 6e

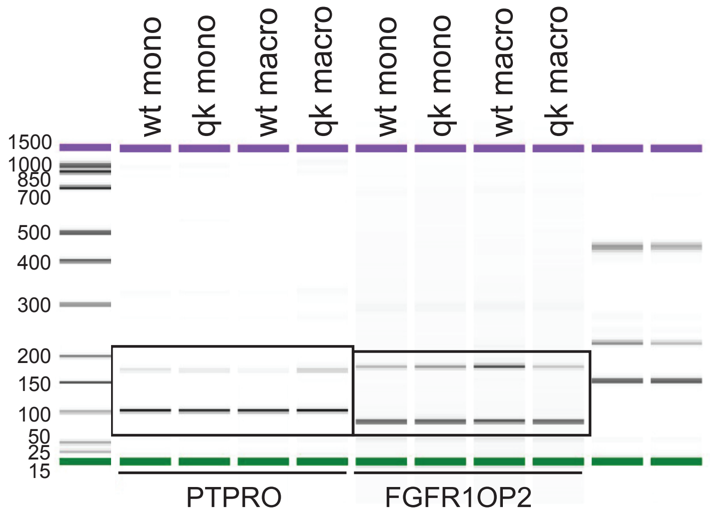

Figure 6e

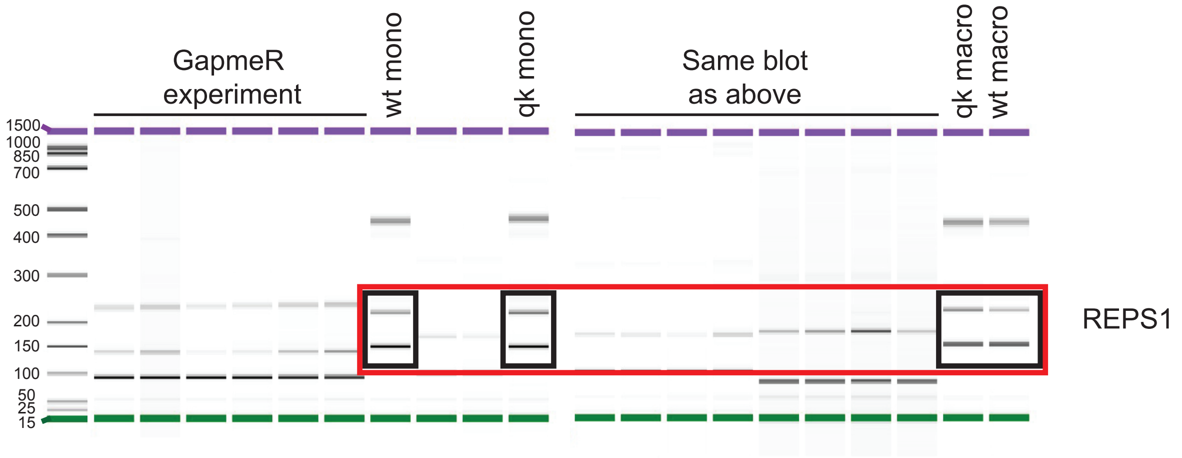

Note: A pipeting miscue in left gel prompted us to load the two remaining samples in another blot.

Supplementary Figure 9: Original bioanalyzer images utilized for preparation of figures 6d and 6e.

Supplementary Figure 10

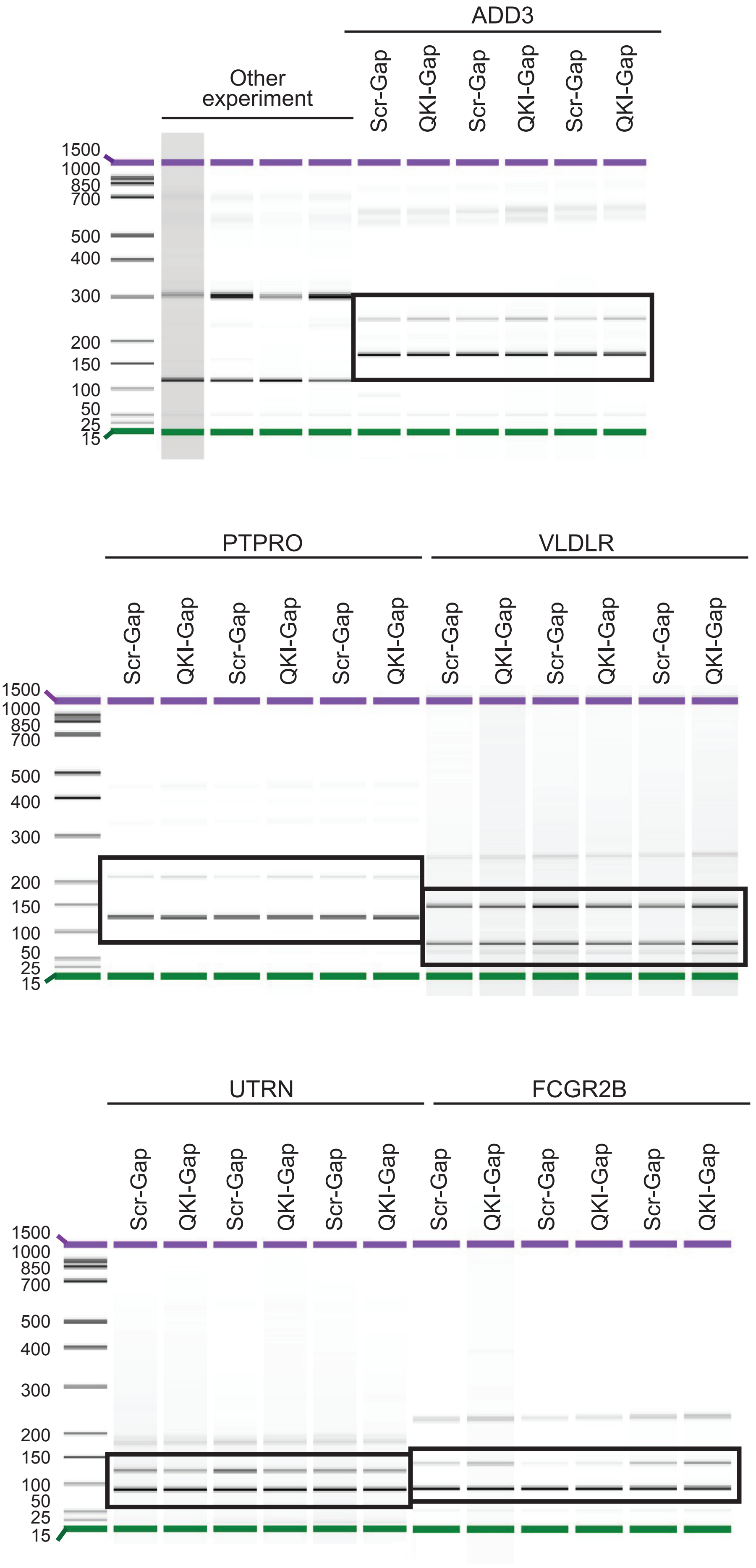

Supplementary Figure 10: Original bioanalyzer images utilized for preparation of figures 4f.

Supplementary Table 1

Transcript abundance primers		5'--> 3'
hsa+mmu-QKI-5	Forward	CTGTCATGCCAAACGGAAC
	Reverse	GATGGACACGCATATCGTG
hsa+mmu-QKI-6	Forward	CTGTCATGCCAAACGGAAC
	Reverse	CGTTGGGAAAGCCATAC
hsa+mmu-QKI-7	Forward	CTGTCATGCCAAACGGAAC
	Reverse	GACTGGCATTTCAATCCAC
hsa-GAPDH	Forward	TTCCAGGAGCGAGATCCCT
	Reverse	CACCCATGACGAACATGGG
**pre-mRNA splicing primers**		5'--> 3'
hsa-ADD3	Forward	ACCAGCTCCTCCTAACCCAT
	Reverse	TCACTCGCTTAGCAAGCTCAT
hsa-ERBB2IP	Forward	AGTTCCTCGTGACTGGAGAGA
	Reverse	AATGGGTTTCCTCTACCCCC
hsa-KIF13A	Forward	GTGCAGCATTCAGGGACACT
	Reverse	GCATCTGACCACCTCTCCCT
hsa-LAIR1	Forward	AAACATTCCGCCTGGAGAGG
	Reverse	CATTGTGACTGTTGTCCGACG
hsa-PTPRO	Forward	GGAGTGTGGAGCTGGTACAT
	Reverse	AGGCATCAAAGTCATCCAGTTG
hsa-FCGR2B	Forward	TCACTGGGATTGCTGTAGCG
	Reverse	GCCTCATCAGGATTAGTGGGA
hsa-UTRN	Forward	TTGCCAAACACCCTCGACTT
	Reverse	AACAGTTGAGGAGATTGTGAGGG
mmu-PTPRO	Forward	ATGTGGAGCTGGCACGTTTG
	Reverse	ACGGGGTTTGTTAGTTTCCTCT
mmu-FGFR1OP2	Forward	CATGGCCAGCAAGAAAGATGAC
	Reverse	TTTGGTCAACATGTGCTTGC
mmu-REPS1	Forward	AGCCAGGTGAGGTAGGTTACT
	Reverse	CTGCATGTGGATTTTGCTTGGA

Atherosclerosis-related genes		5'--> 3'
hsa-MYLIP/IDOL	Forward	CATCTTACAGGAGCAGACTAGGC
	Reverse	TTGGCAGTGTTCTGGTTGTAG
hsa-ABCG1	Forward	ATTCAGGGACCTTTCCTATTCGG
	Reverse	CTCACCACTATTGAACTTCCCG
hsa-ABCA1	Forward	ACCCACCCTATGAACAACATGA
	Reverse	GAGTCGGGTAACGGAAACAGG
hsa-SCARB1	Forward	ACTTCTGGCATTCCGATCAGT
	Reverse	ACGAAGCGATAGGTGGGGAT
hsa-PPARD	Forward	GCCTCTATCGTCAACAAGGAC
	Reverse	GCAATGAATAGGGCCAGGTC
hsa-PPARG	Forward	TACTGTCGGTTTCAGAAATGCC
	Reverse	GTCAGCGGACTCTGGATTCAG
hsa-PPARA	Forward	ATGGTGGACACGGAAAGCC
	Reverse	CGATGGATTGCGAAATCTCTTGG
hsa-NR1H2	Forward	AGAAGATTCGGAAACAACAGCA
	Reverse	GCTGGATCATTAGTTCTTGAGCC
hsa-ApoE	Forward	GTTGCTGGTCACATTCCTGG
	Reverse	GCAGGTAATCCCAAAAGCGAC
hsa-CD36	Forward	CTTTGGCTTAATGAGACTGGGAC
	Reverse	GCAACAAACATCACCACACCA
hsa-LDLR	Forward	ACGGCGTCTCTTCCTATGACA
	Reverse	CCCTTGGTATCCGCAACAGA
hsa-VLDLR	Forward	CTGGGTATGCGACGATGATG
	Reverse	CTTGGTGTGTATGACTGGCTG
hsa-NR1H3	Forward	CTCAATGCCTGATGTTTCTCCT
	Reverse	TCCAACCCTATCCCTAAAGCAA
mmu-PPARD	Forward	TCCATCGTCAACAAAGACGGG
	Reverse	ACTTGGGCTCAATGATGTCAC
mmu-PPARG	Forward	GGAAGACCACTCGCATTCCTT
	Reverse	GTAATCAGCAACCATTGGGTCA
mmu-PPARA	Forward	AGAGCCCCATCTGTCCTCTC
	Reverse	ACTGGTAGTCTGCAAAACCAAA
mmu-NR1H2	Forward	ATGTCTTCCCCCACAAGTTCT
	Reverse	GACCACGATGTAGGCAGAGC
mmu-NR1H3	Forward	CTCAATGCCTGATGTTTCTCCT
	Reverse	TCCAACCCTATCCCTAAAGCAA
mmu-ApoE	Forward	CTGACAGGATGCCTAGCCG
	Reverse	CGCAGGTAATCCCAGAAGC
mmu-CD36	Forward	AGATGACGTGGCAAAGAACAG
	Reverse	CCTTGGCTAGATAACGAACTCTG
mmu-LDLR	Forward	TCAGACGAACAAGGCTGTCC
	Reverse	CCATCTAGGCAATCTCGGTCTC
mmu-VLDLR	Forward	GAGTCTGACTTCGTGTGCAAA
	Reverse	GAACCGTCTTCGCAATCAGGA
mmu-NR1H3	Forward	CTCAATGCCTGATGTTTCTCCT
	Reverse	TCCAACCCTATCCCTAAAGCAA
mmu-MYLIP/IDOL	Forward	ATGCTGTGCTATGTGACGAGG
	Reverse	TCGATGATCCCTAGACGCCTG

Supplementary Table 1: Primers sets utilized to determine mRNA abundance and pre-mRNA splicing events.

Supplementary Reference

1. Gertz et.al. Distinct properties of cell-type-specific and shared transcription factor binding sites. *Mol. Cell* 52, 25-36 (2013).
